# Mechanistic Divergence and Ozone Mass Transfer Enhancement in Photocatalytic Ozonation: A Comparative Study of Bimetallic Pd/Cu-Modified Rutile TiO_2_ and g‑C_3_N_4_

**DOI:** 10.1021/acsomega.6c09130

**Published:** 2026-10-13

**Authors:** Marta Gmurek, Cong Xiaoyu, Jacek Balcerzak, Paweł Mazierski, Magdalena Miodyńska-Melzer, Tomasz Klimczuk, Adriana Zaleska-Medynska, Dominik Knozowski, Radian Popescu, Thomas Schwartz, Harald Horn

**Affiliations:** † Institute of General and Ecological Chemistry, Faculty of Chemistry, 49584 Lodz University of Technology, Zeromskiego 116, Lodz 90-924, Poland; ‡ Institute of Functional Interfaces (IFG), 28335 Karlsruhe Institute of Technology (KIT), Hermann von Helmholtz Platz 1, Eggenstein-Leopoldshafen 76344, Germany; § Engler-Bunte-Institut, Water Chemistry and Water Technology, 150232 Karlsruhe Institute of Technology, Engler-Bunte-Ring 9a, Karlsruhe 76131, Germany; ∥ Division of Molecular Engineering, Faculty of Process & Environmental Engineering, 49584 Lodz University of Technology, Wolczanska 213, Lodz 93-005, Poland; ⊥ Department of Environmental Technology, Faculty of Chemistry, 427055 University of Gdansk, Wita Stwosza 63, Gdansk 80-308, Poland; # Department of Solid State Physics, Faculty of Applied Physics and Mathematics, Gdansk University of Technology, Narutowicza 11/12, Gdansk 80-233, Poland; ∇ Laboratory for Electron Microscopy, Karlsruhe Institute of Technology KIT, Finanzmanagement Kaiserstraße 12, Karlsruhe D-76131, Germany; ○ DVGW German Technical and Scientific Association for Gas and Water Research Laboratories, Water Chemistry and Water Technology, Engler-Bunte-Ring 9a, Karlsruhe 76131, Germany

## Abstract

Beyond conventional removal-efficiency metrics, this investigation reveals a mechanistic divergence between two prominent photocatalysts in ozone activation. Both (Pd/Cu) bimetallic g-C_3_N_4_ and rutile TiO_2_ enable efficient antibiotic degradation and antibiotic resistance genes inactivation; however, they rely on fundamentally different reaction pathways. (Pd/Cu)^1%^C_3_N_4_ predominantly generates reactive oxygen species (ROS), including singlet oxygen (^1^O_2_) and superoxide radical anion (O_2_^●–^), whereas (Pd/Cu)^1%^TiO_2_ targets specific sequences like *intl*1 bypassing conventional ROS pathways. In addition, our research highlights a critical design trade-off and operational paradox in advanced oxidation processes: while a low bimetallic loading (0.1 wt %) optimizes the initial chemical degradation kinetics of both antibiotics and antibiotic resistance genes during active treatment, a higher loading (1.0 wt %) is strictly indispensable for suppressing long-term bacterial regrowth and preventing the biological resuscitation of resistance markers during post-treatment storage. Moreover, this study challenges conventional methodological paradigms by demonstrating that diagnostic scavengers are not inert probes but reactive species that accelerate ozone (O_3_) depletion and alter mass-transfer behavior, potentially confounding mechanistic interpretations. In complex wastewater matrices dominated by dissolved organic matter, such competitive interactions necessitate rigorous scavenger validation to ensure reliable translation of laboratory findings to scalable purification systems.

## Graphical abstract

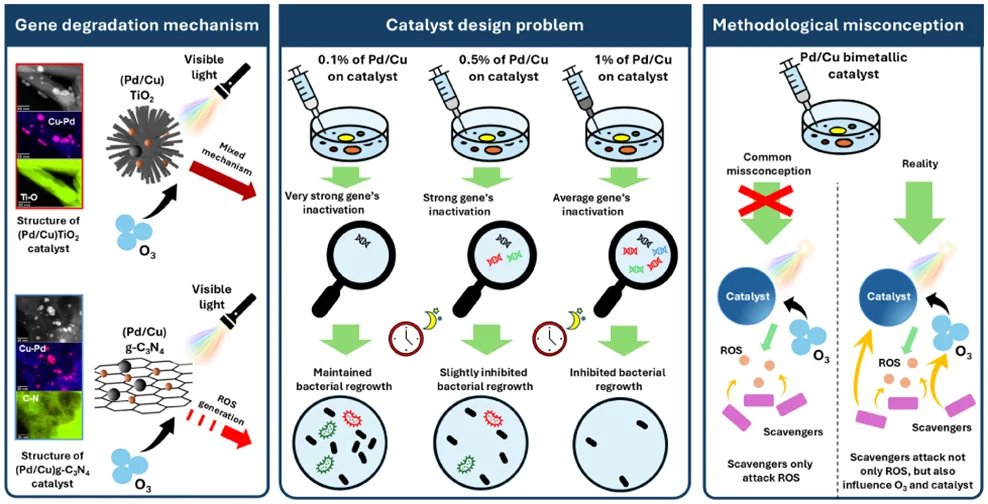

## Introduction

In recent years, there is a rising concern regarding the quality of water and the efficacy of the currently used water treatment procedures. It is estimated that only 63% of the world’s wastewater is collected, even excluding agricultural runoff, while 52% of wastewater is treated at all.^1^ In addition, there is a growing problem of so-called contaminants of emerging concern (CEC), pollutants which are hard to remove by conventional water treatment facilities. This group includes multiple substances such as hormones, personal care products, pesticides, antibiotics, and antibiotic resistance genes (ARGs). ARGs are especially dangerous because by incorporating antibiotic traits into bacteria at the gene level, they directly influence the effectiveness of medical therapies.^2^ In the EU summary report (2022–2023) it was shown that due to the presence of ARGs in wastewater, hazardous pathogens like *E. coli* bacteria started to develop antibiotic resistance, including resistance to critically important antimicrobials (CIAs).^3^ Therefore, there is a great need to limit the concentration of both antibiotics and ARGs in discharged effluents.

To combat this problem, multiple strategies have been widely developed. Recent reports show a great interest in the utilization of advanced oxidation processes (AOPs) for CEC removal.^4^ AOPs include various techniques like Fenton and Fenton-like processes, electro-peroxone, anodic oxidation, photocatalysis, and ozonation.^5^ Depending on the selected method and wastewater characteristics, different reactive oxygen species (ROS) like superoxide radicals (O_2_^●–^), hydroxyl radicals (^●^OH), hydrogen peroxide (H_2_O_2_), or singlet oxygen (^1^O_2_) play a decisive role in water decontamination.^5,6^ However, although AOPs in general prove to be very effective in antibiotic degradation, there are two main worrying aspects: *i*) there are unknown toxicity characteristics of mineralization intermediates and byproducts that can be more hazardous than the initial contaminant, and *ii*) AOPs may, in some cases, introduce ARGs into pathogenic bacteria.^4^ Thus, selecting the appropriate AOP method for a specific wastewater is a key issue in the water treatment process.

Photocatalytic ozonation, an innovative AOP process that merges photocatalysis and ozonation, has garnered significant attention as a potent technique for degrading organic contaminants in water. The prominent aspect of this technique is its high efficiency in pollutant degradation with a limited amount of byproducts compared to traditional water treatment methods. In the typical photocatalytic ozonation process, a photon is absorbed by the photocatalyst, which leads to electron–hole separation. Then, ozone absorbs these newly created electrons/holes, and, after a series of reactions, produces various ROS.^7–9^ The key parameters influencing this process are an appropriate band gap, a low recombination rate, and ozone and pollutant adsorption capabilities.^7,9^ Therefore, among the plethora of available photocatalytic materials, titanium dioxide (TiO_2_) and graphitic carbon nitride (g-C_3_N_4_ or simply C_3_N_4_) have emerged as frontrunners due to their exceptional photocatalytic properties. Both materials exhibit different decontamination behavior while used in the photocatalytic ozonation process. The photocatalytic activity of TiO_2_ primarily involves the generation of electron–hole pairs upon light absorption with simultaneous adsorption of ozone molecules, followed by redox reactions that lead to the formation of surface-bound ozone species, which are later transformed into ROS. These interactions result in large amounts of ROS in the form of hydroxyl radicals, bound hydroxyl radicals, and/or positive holes. The ozone species and newly created ROS can react with organic pollutants or participate in redox reactions, contributing to the degradation process.^6,10^ The degradation activity depends on the crystal structure of TiO_2_, and it is believed that although rutile generates more hydroxyl radical species, they are strongly bound to the catalyst, leading to overall poorer performance.^6^ On the other hand, C_3_N_4_, due to the higher-lying conduction band (−1.3 V in comparison to −0.1 V vs NHE for C_3_N_4_ and TiO_2_, respectively), exhibits a different reaction pathway in photocatalytic ozonation. When exposed to ozone molecules, first O_2_^●–^ species are formed, which consequently react with O_3_ causing formation of O_3_^●–^ which further are transformed into hydroxyl radicals; in addition, during photocatalytic oxidation, ^●^OH are formed from O_2_^●–^ and H_2_O_2_.^11,12^ All of these ROS play a crucial role in oxidizing organic pollutants, facilitating their degradation into harmless byproducts.

Despite their differences in ROS production, both catalysts share some similarities. TiO_2_ and C_3_N_4_ are known for their reasonable cost, abundance, chemical photostability, semiconductive character, and the possibility to tailor their properties according to needs.^13–15^ Unfortunately, both materials are also characterized by high recombination rates of electrons/holes. In addition, in the case of TiO_2_, the band gap is ∼3.2 eV,^8^ which limits the utilization of sunlight to ∼5% (the percentage of UV in the sunlight spectrum reaching Earth’s surface^16^). On the other hand, C_3_N_4_ possesses a high-laying valence band which hinders its catalytic activity.^17^ Therefore, strategies like doping with foreign metals have been adopted to enhance the both of these materials’ photocatalytic activity. Previous works describe the successful construction of various composites for photocatalytic oxidation like ZnO/TiO_2_,^18^ Pd/TiO_2_,^19^ WO_3_/TiO_2_,^20^ and Ag/C_3_N_4_.^21^ Palladium doping is particularly interesting because it exhibits a lower reduction potential than platinum, and at the same time favors losing two electrons in contrast to silver or gold.^22^ However, palladium, as a noble metal, is rare and expensive, and therefore there is a movement toward bimetallic systems, like palladium–copper (Pd/Cu). Pd/Cu is especially attractive, as synergistic and cooperative interactions can be tailored through manipulation of the palladium/copper ratio. Moreover, this system exhibits excellent redox properties thanks to the electron-donor properties of Cu and the electron-acceptor character of palladium, and thus Pd/Cu has been applied for numerous applications.^23^ In our previous work,^24^ we presented for the first time the great potential of Pd/Cu-TiO_2_ for ARG and pathogenic bacteria removal, as well as improvement of quantum efficiency, lower recombination rates, and good stability. However, that research concerned only TiO_2_ modification. As both C_3_N_4_ and TiO_2_ exhibit different interactions with ozone during photocatalytic ozonation, understanding the impact of the Pd/Cu additive is essential for optimizing the design of photocatalytic ozonation systems tailored to specific water treatment applications.

In this work, we compared the effect of modification of TiO_2_ and C_3_N_4_ by bimetallic Pd/Cu for both antibiotic and ARG removal. To obtain full information, we studied both ARGs and integron markers as well as rDNA in secondary effluent. The aim of this study was to elucidate the mechanistic divergence and ozone mass-transfer behavior in photocatalytic ozonation systems employing bimetallic Pd/Cu-modified TiO_2_ and C_3_N_4_. Specifically, the study sought to *i*) compare the pathways of ozone activation and pollutant transformation; *ii*) assess the role of reactive oxygen species versus non-ROS mechanisms in the degradation of antibiotics and ARGs; *iii*) evaluate the relationship between catalyst loading, degradation kinetics, and post-treatment biological inactivation; and *iv*) critically examine the influence of scavenger compounds and dissolved organic matter on ozone consumption and mechanistic interpretation under environmentally relevant conditions. This work presents the first comprehensive mechanistic comparison of bimetallic Pd/Cu-modified rutile TiO_2_ and C_3_N_4_ for the photocatalytic ozonation of antibiotics and resistance genes in complex wastewater matrices. Challenging standard mechanistic assumptions, we demonstrate that radical scavengers are not passive probes but active participants that fundamentally alter ozone decomposition kinetics, necessitating a reevaluation of ROS roles via ozone consumption monitoring. The investigation reveals a distinct mechanistic divergence where C_3_N_4_ relies predominantly on ROS-driven pathways for gene inactivation, whereas TiO_2_ exhibits a unique resistance to scavenging effects, suggesting a direct charge-transfer mechanism for recalcitrant targets like *intl*1. Furthermore, we identify a critical operational trade-off, establishing that while low metal loadings (0.1%) optimize initial degradation rates, higher loadings (1%) are indispensable for preventing post-treatment bacterial regrowth.

## Experimental Section

### Catalyst Synthesis and Preparation

Synthesis of pristine and bimetallic Pd/Cu–TiO_2_ and Pd/Cu–C_3_N_4_ materials: Pristine rutile TiO_2_ with an urchin-like morphology and the C_3_N_4_ materials were synthesized following the procedure reported in our previous work.^25–27^ Bimetallic Pd/Cu nanoparticles were photodeposited on TiO_2_ and C_3_N_4_ in a quartz photoreactor at 10 °C using potassium tetrachloropalladate (II) and copper (II) diacetate monohydrate as precursors. The Pd/Cu modifier was obtained using a nominal 1:1 weight precursor ratio of Pd to Cu, which corresponds to a theoretical atomic precursor ratio of approximately 1:1.67 (Pd:Cu). The actual spatial distribution and surface enrichment deviate from this bulk stoichiometry due to depth-segregated nucleation, as detailed in our morphological characterization. The photocatalyst, ethanol, and metal precursors were mixed, stirred under nitrogen, irradiated for 1 h, then repeatedly washed and dried, with Pd/Cu loading controlled by the precursor amount. Further experimental details are provided in ref^27^.

### Catalyst Characterization

Material characterization included surface morphology by field-emission scanning electron microscopy (FE-SEM, JEOL JSM-7610F), crystal structure by powder X-ray diffraction (Bruker D8 Advance Eco, Cu Kα, LynxEye-XE detector, Le Bail method with HighScore Plus software), chemical structure by Raman spectroscopy (Thermo Scientific DXR Smart Raman, 532 nm laser), and optical properties and XPS quantification analysis by UV–Vis diffuse reflectance spectroscopy (UV-Vis/DRS) (UV-2600, Shimadzu; BaSO_4_ reference). Photoluminescence (PL) spectra were recorded at room temperature using a PerkinElmer LS-50B luminescence spectrometer equipped with a xenon lamp and an R928 photomultiplier, with an excitation wavelength of 280 nm.

Transmission electron microscopy (TEM), high-angle annular dark-field scanning TEM (HAADF-STEM), and energy-dispersive X-ray spectroscopy (EDXS) were performed on an FEI Tecnai Osiris microscope operating at 200 keV and fitted with a Super-X EDXS system with four silicon drift detectors. Samples for HAADF-STEM/EDXS were prepared by drop-casting diluted nanoparticle suspensions onto ultrathin (3 nm) amorphous carbon films on holey carbon supports mounted on 400-mesh Au grids.

Average elemental composition of the nanoparticles was obtained from EDXS area scans, using Ti–K, Cu–K, Pd–L, and O–Kα lines for TiO_2_-based samples, and Cu–K, Pd–L, C–Kα, and N–Kα lines for C_3_N_4_-based samples. Samples containing 0.5% and 1.0% Pd/Cu on TiO_2_ and C_3_N_4_ were further examined by X-ray photoelectron spectroscopy (XPS; Kratos AXIS Ultra, monochromatic Al Kα, 1486.6 eV), using a 300 × 700 μm analysis area, 150 W anode power, and a pass energy of 20 eV for high-resolution scans; a charge neutralizer was applied, and the main adventitious carbon peak was used to calibrate the binding energy scale. The specific surface area was measured using the Brunauer–Emmett–Teller (BET) method in the relative pressure range (p/p_0_) of 0.05–0.3, employing a Micro 200 analyzer (3P Instruments).

### Catalytic Activity: Photocatalytic Ozonation Experiments

All reactions were carried out in a 500 cm^3^ glass reactor with continuous magnetic stirring. The reactor was placed inside an Oriel Sol3A Class AAA solar simulator (Newport Corp., Irvine, CA) equipped with a 1000 W xenon arc lamp with a UV cutoff, providing visible light in the 350–800 nm range; the incident photon flux was 448 W/m^2^ as measured with an Ocean Optics USB4000 fiber-optic spectrometer. Ozone was produced in situ by an ozone generator (803 N, BMT) generated from a pure oxygen stream. The gas was continuously bubbled and monitored at the reactor inlet and outlet by O_3_ analyzers (363, BMT), while residual ozone in the exhaust gas was captured in a potassium iodide (KI) solution. The gas flow rate and the inlet ozone concentration were maintained at 0.4 Ndm^3^/min and 37 mgO_3_/Ndm^3^, respectively. The mass transfer coefficient *k* *_L_a* was determined as 0.72 1/min, which is in agreement with the similar values reported by ref^28^, for secondary effluent. Each photocatalytic ozonation experiment employed metal-doped TiO_2_ (rutile) and C_3_N_4_ nanostructures at a catalyst loading of 150 mg/dm^3^. Suspensions were equilibrated for 15 min in the dark before irradiation to assess catalyst-only effects, followed by 60 min of treatment. The comprehensive experimental matrix, detailing the baseline control runs (including light-only, ozone-only, and dark adsorption experiments) alongside the combined catalytic treatments, is documented in Table S1 of the Supporting Information. Bacterial regrowth was assessed for all trials by storing 200 cm^3^ treated effluent in the dark at room temperature for 72 h post-treatment.

### Environmental Sampling and Analysis

This study evaluates photocatalytic ozonation efficacy for inactivating antibiotic resistance genes (ARGs: *bla*_TEM_, *erm*B, *qnr*S, *tet*_M_), the class 1 integron marker (*intl*1), eubacterial 16S rDNA, and enterococcal 23S rDNA in secondary effluent. Samples were collected weekly from the Karlsruhe-Neureut WWTP (Germany), with ARG/ARB quantification, DNA extraction, and quantitative polymerase chain reaction (qPCR) protocols detailed in our prior publications.^24,27^ Additionally, differences in the photocatalytic ozonation mechanism were investigated toward sulfamethoxazole (SMX) degradation. SMX concentrations were measured using an Agilent 1290 Infinity II UHPLC system coupled with an Agilent 6470 Triple Quadrupole LC/MS. The full methodology for this analysis is described in our previously published paper.^29^ To validate the environmental significance of the qPCR results, the reduction in amplifiable ARG copies was correlated with the loss of actual biological transforming activity. As demonstrated in our previous horizontal gene transfer (HGT) and bacterial conjugation assays under comparable bimetallic solar-AOP conditions,^24,27^ a reduction in amplifiable gene copies by qPCR directly corresponds to a near-complete loss (>99%) of biological transforming capability.

## Results and Discussion

Various techniques, including XRD, SEM, TEM, photoluminescence (PL), X-ray photoelectron spectroscopy (XPS), EDX, UV–Vis/DRS and Raman, were employed to assess the optical characteristics, structural morphology, and chemical compositions. The obtained results for these techniques are elaborated upon in the following sections. The catalytic activity of catalysts has been investigated toward ARGs inactivation as well as sulfamethoxazole removal. The mechanism of action has been also checked leading to find the differences between investigated catalysts.

### Catalyst Characterization

#### Optical Characteristics

The PL emission spectra of synthesized bimetallic (Pd/Cu)­TiO_2_ and (Pd/Cu)­C_3_N_4_ photocatalysts are presented in Figure 1. Pristine C_3_N_4_ and TiO_2_ photocatalysts exhibited characteristic PL emission spectra.^30,31^ In the case of modified C_3_N_4_ and TiO_2_, the intensity of the PL emission spectrum is reduced compared with the pristine materials. The observed intensity variations among the different samples provided valuable insights into the influence of Pd/Cu loading. The lowest PL emission intensity observed for the (Pd/Cu)^0.5%^C_3_N_4_ and (Pd/Cu)^0.1%^TiO_2_ samples suggests that these samples exhibit the most favorable conditions for effective charge carrier separation and utilization.^32,33^ Thus, the optimized Pd/Cu loading concentration appears to enhance the photocatalytic activity by facilitating light absorption and the transfer of photoexcited charges and reducing their recombination, leading to improved overall performance. Moreover, Figure 1 shows the diffuse reflectance spectra of synthesized bimetallic (Pd/Cu)­TiO_2_ and (Pd/Cu)­C_3_N_4_ photocatalysts. Pristine TiO_2_ exhibited strong absorption below 420 nm due to its rutile nature,^34^ while pristine C_3_N_4_ absorbed most efficiently in the 250–460 nm range, consistent with its 2.7 eV band gap.^35^ Modified samples extended absorption into the visible range (above 420 nm for TiO_2_ and above 480 nm for C_3_N_4_) due to the presence of Pd and Cu nanoparticles on the photocatalyst surface.^36,37^ In both cases, the absorption intensity increased with the amount of deposited bimetallic Pd/Cu nanoparticles, indicating enhanced light absorption and potentially improved photocatalytic properties.

**Figure 1:**
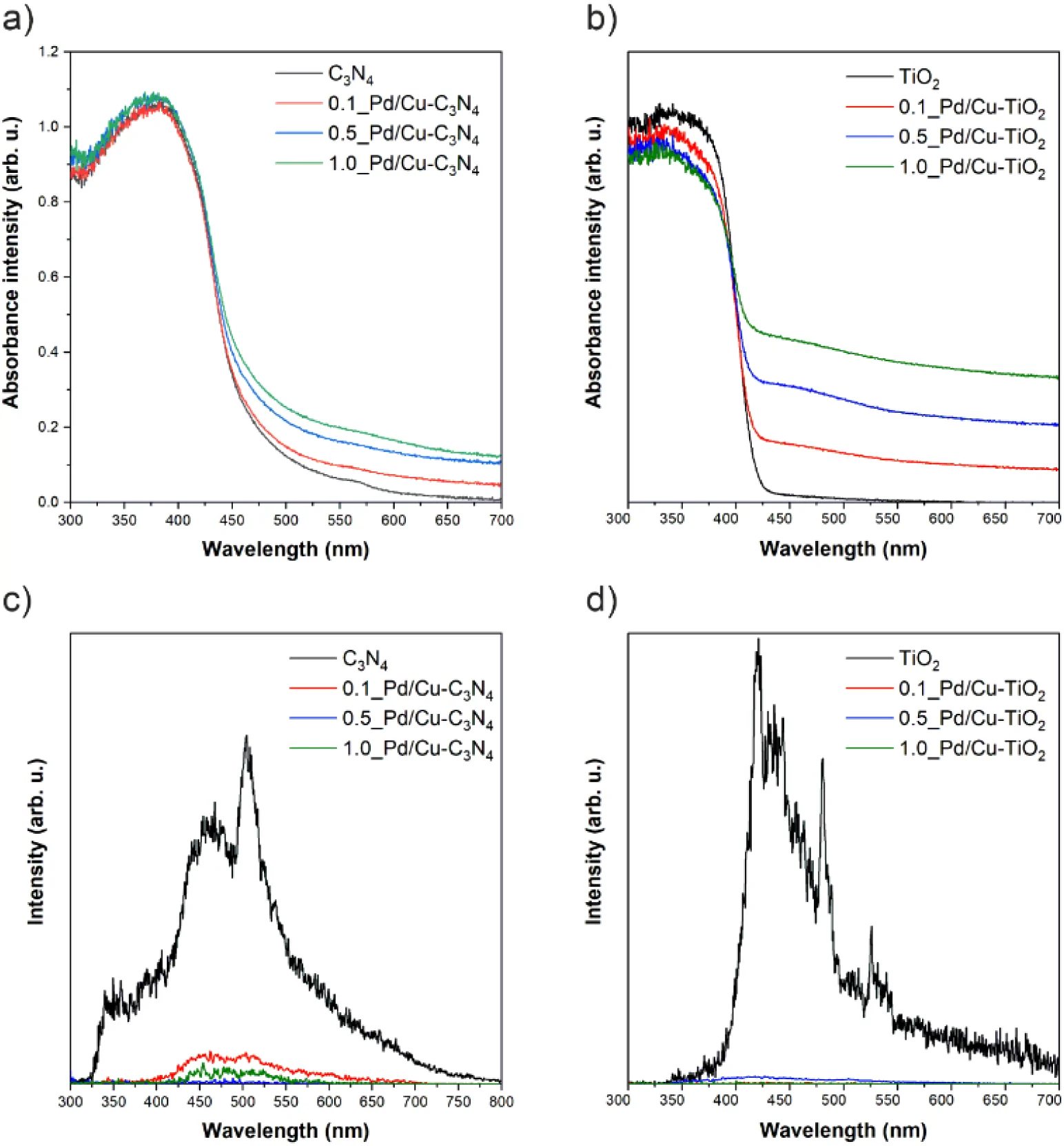
DRS UV–Vis and PL emission spectra of a, c) (Pd/Cu)­C_3_N_4_ and b, d) (Pd/Cu)­TiO_2_ photocatalysts.

### Structural and Morphological Properties

XRD patterns of both pristine and bimetallic (0.1, 0.5, and 1.0% (Pd/Cu)­TiO_2_ and (Pd/Cu)­C_3_N_4_) photocatalysts are shown in Figure 2. In all synthesized samples, one can observe diffraction peaks originating from TiO_2_ (Figure 2a) and C_3_N_4_ (Figure 2b), while reflections from bimetallic Pd/Cu nanoparticles were not noticed probably due to their insufficient amount. In a series of Pd/Cu-modified TiO_2_ samples, a strong signal was recorded at 2θ = 27.3°, while reflections of lower intensity were present at 2θ = 36.0°, 39.1°, 41.1°, 44.0°, 54.2°, 56.6°, 62.7°, 64.0°, and 69.0°. The diffraction peaks listed above can be attributed to (110), (101), (200), (111), (210), (211), (220), (002), (310), and (112) planes,^38^ respectively, which confirms the formation of crystalline rutile.^39^ In the case of pristine and modified C_3_N_4_, two reflections corresponding to the (100) and (002) planes were recorded at 2θ = 13.1°, 27.2°,^40,41^ respectively. Raman spectra presented in Figure 2c,d confirmed the above XRD results. Bands observed in TiO_2_-based samples located at 143, 238, 443, and 608 cm^–1^ are observed due to the rutile phase of TiO_2_.^42^ Raman spectra of C_3_N_4_ samples consisted of several bands in the range of 700–1600 cm^–1^, which correspond well to the structure of graphitic carbon nitride.^43,44^ It can be seen that modification of the TiO_2_ and C_3_N_4_ surfaces with Cu and Pd NPs did not introduce visible changes in the semiconductors’ structures.

**Figure 2:**
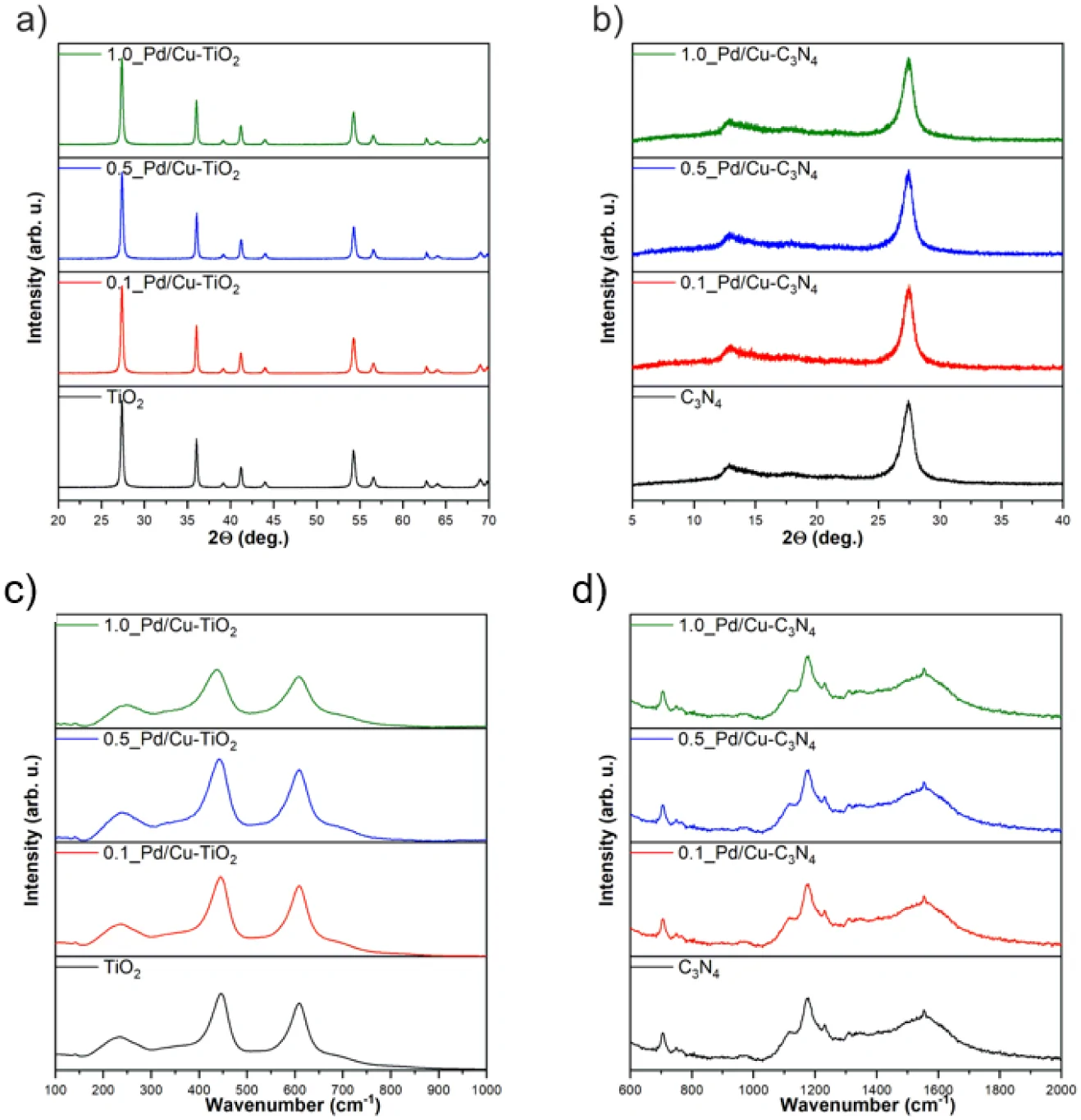
XRD patterns and Raman spectra of a, c) (Pd/Cu)­TiO_2_ and b, d) (Pd/Cu)­C_3_N_4_ photocatalysts, respectively.

The morphology of bimetallic-modified (Pd/Cu)^1%^TiO_2_ and (Pd/Cu)^1%^C_3_N_4_ photocatalysts is presented in Figure S1. Rutile-modified TiO_2_ presents elongated crystalline nanorods (which can be seen in Figure 3a,c as well), which are closely packed and possess square profiles, while modified C_3_N_4_ exhibits an irregular flake-like morphology. The HAADF-STEM images reveal a distinct contrast difference between the quasi-spherical particles, characterized by heavier Cu and Pd nanoparticles, displaying a bright contrast, and the elongated TiO_2_ particles, which consist of lighter Ti and O elements, exhibiting a dark contrast (Figure 3a,c). Similarly, the images depict a clear contrast disparity between the large C_3_N_4_ particles, composed of light C and N elements, showing a dark contrast (Figure 3b,d). The EDXS mapping of Ti–O reveals a uniform distribution of Ti and O within the elongated nanoparticles, as observed in the HAADF-STEM image. Conversely, the EDXS map of Cu and Pd illustrates the presence of quasi-spherical particles, primarily composed of a Pd-rich Pd–Cu alloy with most of the Pd contained within these particles. In contrast, Cu is mainly distributed on the surface of the elongated TiO_2_ particles, forming small Cu–Pd particles. Similarly, in the case of C_3_N_4_, the EDXS map of C–N displays a uniform distribution of carbon (C) and nitrogen (N) within large support nanoparticles. The EDXS map of Cu and Pd indicates the formation of particles primarily composed of a Pd-rich Pd–Cu alloy, with most of the Pd contained within these particles, while Cu is predominantly distributed on the surface of the C_3_N_4_ support particles.

**Figure 3:**
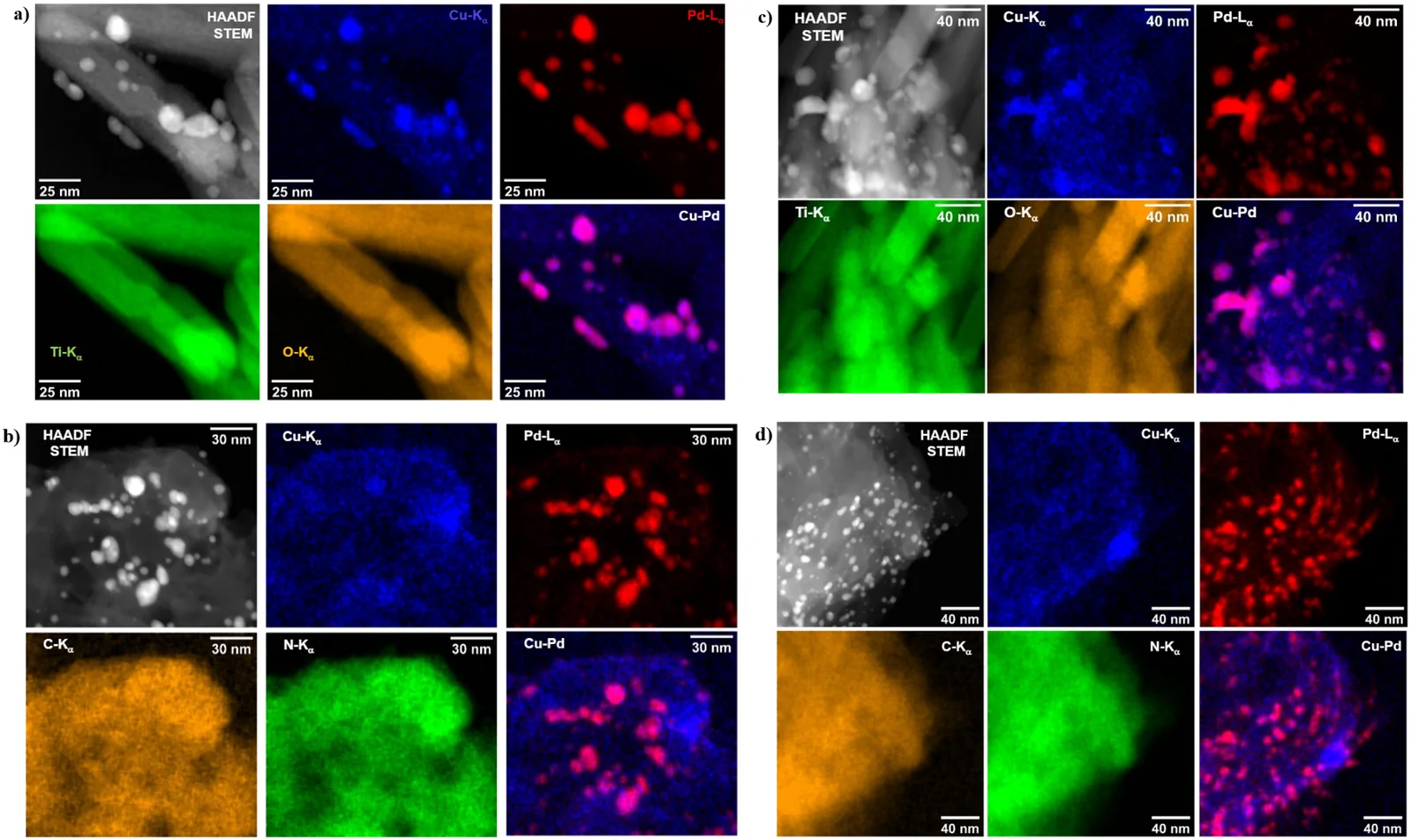
Structural characterization of different supported Pd/Cu catalysts by HAADF-STEM and the corresponding EDXS elemental maps with size distributions of a) (Pd/Cu)^1%^TiO_2_, b) (Pd/Cu)^1%^C_3_N_4_, c) (Pd/Cu)^0.5%^TiO_2_, and d) (Pd/Cu)^0.5%^C_3_N_4_ photocatalysts.

Additionally, fine distributions of very small Cu–Pd particles are observed on the C_3_N_4_ surface, comprising mainly a Cu-rich Pd–Cu alloy. For both ^1%^Pd/Cu catalysts, the Pd composition increases (Cu composition decreases) with increasing the particle size. The average diameter is slightly higher for the (Pd/Cu)^1%^TiO_2_ catalysts (11.1 ± 6.8 nm); however, it is still comparable to (Pd/Cu)^1%^C_3_N_4_ (9.6 ± 3 nm) (Figure 4a). However, the (Pd/Cu)^1%^TiO_2_ particles (21 nm Pd_93 ± 1_Cu_7 ± 1_; 2.6 nm Pd_51 ± 3_Cu_49 ± 2_) are richer in Pd than (Pd/Cu)^1%^C_3_N_4_ (21 nm Pd_74 ± 1_Cu_26 ± 1_; 3 nm Pd_44 ± 3_Cu_56 ± 2_) (Figure S2). In contrast, the average diameter of the measured particles is much lower in the case of (Pd/Cu)^0.5%^C_3_N_4_ (6.8 ± 2.0 nm) than (Pd/Cu)^0.5%^TiO_2_ (15.6 ± 6.4 nm) (Figure 4b). Moreover, in the case of (Pd/Cu)^0.5%^TiO_2_, the distribution pattern is similar to the (Pd/Cu)^1%^TiO_2_ (Figure 4a), while the Cu and Pd compositions do not change as a function of particle size when (Pd/Cu)^0.5%^C_3_N_4_ is considered (the average composition of these particles is given by Cu_15 ± 4_Pd_85 ± 4_, Figure S2).

**Figure 4:**
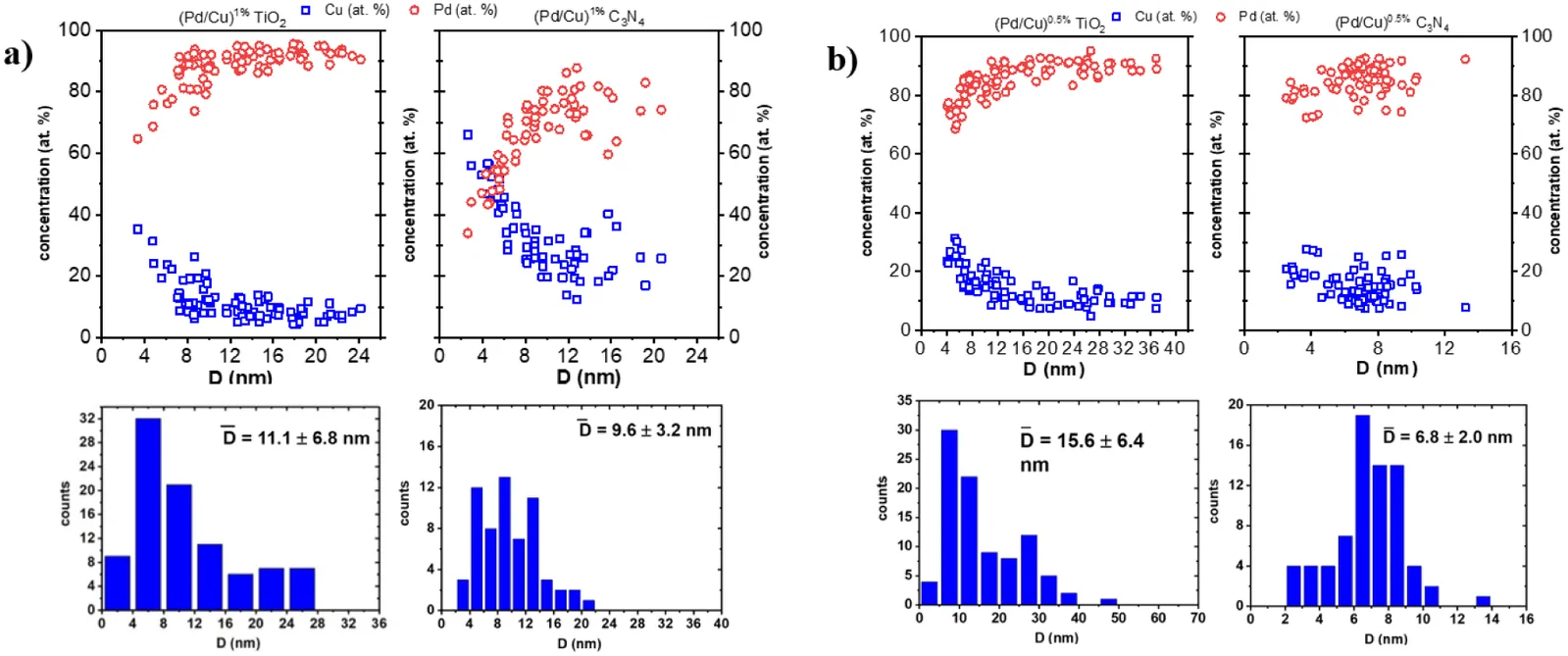
Representative EDX concentration profiles of Cu and Pd nanoparticles and their corresponding particle size distributions of a) (Pd/Cu)^1%^TiO_2_ and (Pd/Cu)^1%^C_3_N_4_ and b) (Pd/Cu)^0.5%^TiO_2_ and (Pd/Cu)^0.5%^C_3_N_4_.

Further characterization was conducted by means of X-ray Photoelectron Spectroscopy. Due to the intrinsic physical detection limits of XPS (typically ∼0.1 at.% for trace transition metals), catalysts prepared with an ultralow nominal loading of 0.1 wt % metal were excluded from XPS analysis. At these trace levels, the signal-to-noise ratio is insufficient to yield statistically reliable deconvolution of Cu 2p and Pd 3d peaks. The structural, optical, and morphological properties of these highly active 0.1 wt % catalysts were instead confirmed using Field Emission Scanning Electron Microscopy (FE-SEM) and Diffuse Reflectance Spectroscopy (DRS). XPS quantification analysis results for 0.5 and 1% of the PD/Cu loading are presented in Table 1 and Table 2 for (Pd/Cu)­TiO_2_ and (Pd/Cu)­C_3_N_4_ samples, respectively. As can be noted, there are approximately equal copper and palladium ion quantity in (Pd/Cu)­TiO_2_ samples (around 0.4% at. for (Pd/Cu)^0.5%^TiO_2_ and 0.9 at.% for (Pd/Cu)^1%^TiO_2_), whereas for catalysts based on C_3_N_4_ the concentration of palladium is greater than copper, i.e. Pd:Cu atomic ratios are equal to 3.0:1 and 2.2:1 for (Pd/Cu)^0.5%^C_3_N_4_ and (Pd/Cu)^1%^C_3_N_4_ samples, respectively. XPS quantitative analysis leads to an information about the composition of the photocatalyst substrates, as well. The oxygen-to-titanium ratio is approximately 2.3:1 for titanium-based samples (Table 1.), and based on this value and on the peak positions in the Ti 2p XPS spectra (not shown), it can be concluded,that the substrate is in the form of TiO_2_ with an addition of small amount of oxygen atoms bonded to adventitious carbon present on the sample surface. Regarding C_3_N_4_-based samples, XPS quantitative analysis results show that there is an excess quantity of carbon atoms in relation to nitrogen (Table 2). The theoretical ratio should be equal to 0.75:1 for C_3_N_4_, whereas it is approximately 0.9:1 and 1:1 for (Pd/Cu)^0.5%^C_3_N_4_ and (Pd/Cu)^1%^C_3_N_4_ samples, respectively. These results lead to the conclusion that there is around 15–24 at.% adventitious carbon within the total carbon quantity present on the sample surface, which is comparable with the carbon concentration revealed on the (Pd/Cu)­TiO_2_ sample surface (Table 1).

**Table 1:** Elemental Composition of (Pd/Cu)­TiO_2_ Samples (XPS Analysis)

Catalyst	O [at.%]	C [at.%]	Ti [at.%]	Pd [at.%]	Cu [at.%]	Pd:Cu
(Pd/Cu)^0.5%^TiO_2_	58.78	15.14	25.31	0.41	0.35	**1.2**
(Pd/Cu)^1%^TiO_2_	56.93	17.15	24.15	0.90	0.88	**1.0**

Elemental XPS spectra of copper (Cu 2p) for (Pd/Cu)­TiO_2_ and (Pd/Cu)­C_3_N_4_ samples are depicted in Figure 5. The characteristic Cu 2p_3/2_ band was deconvoluted into three symmetric components, which are reflected in the Cu 2p_1/2_ band as well. The most intense one centered at 932.1–932.7 eV can be assigned to the Cu^+^ oxidation state, whereas the two others, at positions of 933.8–933.9 eV and 935.1–935.2 eV, represent Cu^2+^ speciation (c1, c2 components).^45,46^ It can be seen that the concentration of Cu^+^ is noticeably higher for C_3_N_4_-based samples (80%) (Figure 5b,d), in comparison with TiO_2_-based ones (70%) (Figure 5a,c), which leads to the conclusion that copper seems to be more reduced on the carbon nitride substrate than it is for the TiO_2_ one Table 2.

**Table 2:** Elemental Composition of (Pd/Cu)­C_3_N_4_ Samples (XPS Analysis)

Catalyst	O [at.%]	C [at.%]	N [at.%]	Pd [at.%]	Cu [at.%]	Pd:Cu
(Pd/Cu)^0.5%^C_3_N_4_	5.20	43.83	50.06	0.68	0.23	3.0
(Pd/Cu)^1%^C_3_N_4_	6.86	45.76	46.14	0.86	0.39	2.2

**Figure 5:**
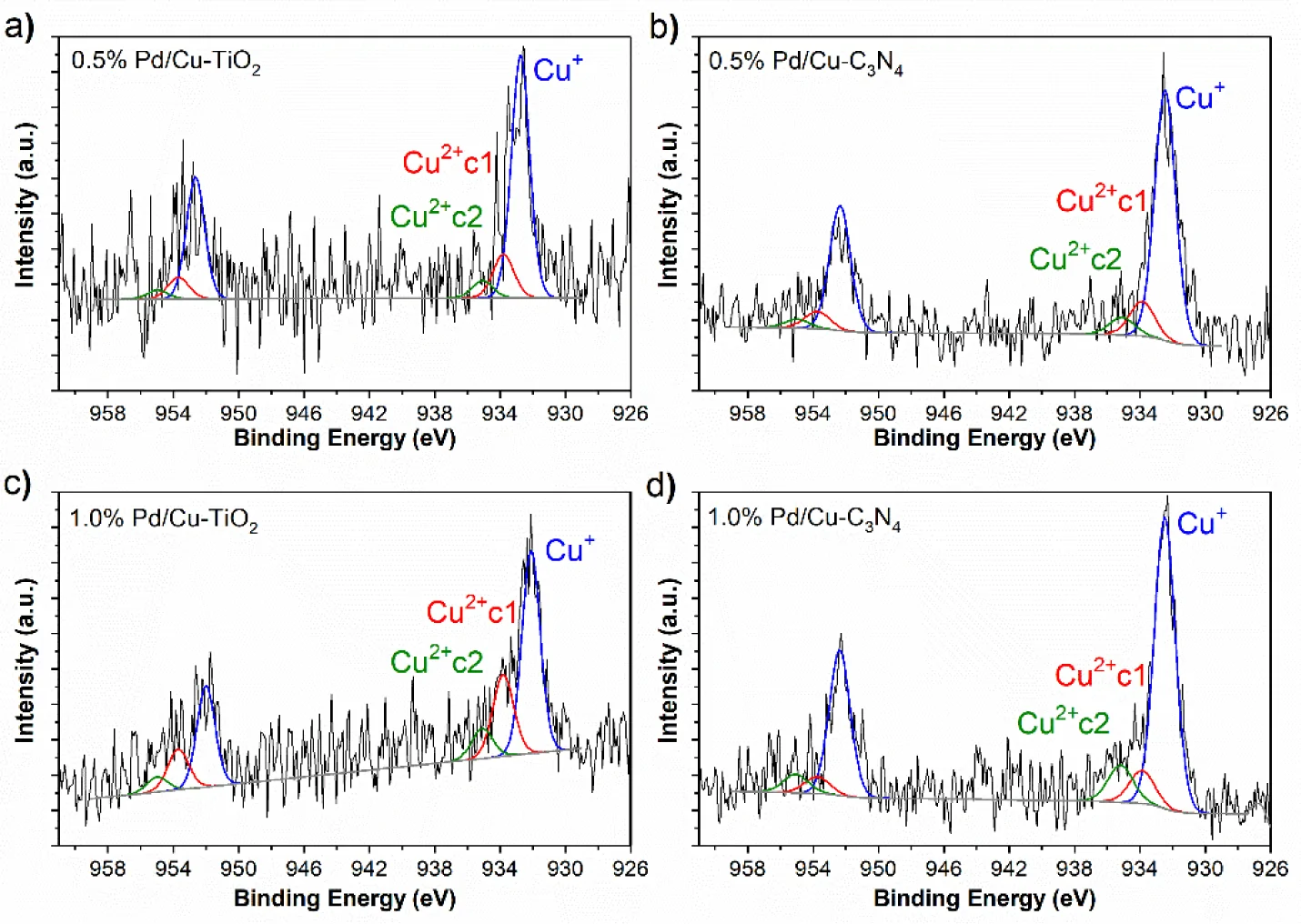
Copper Cu 2p XPS spectra of a, c) (Pd/Cu)­TiO_2_ and b, d) (Pd/Cu)­C_3_N_4_ photocatalysts.

Palladium Pd 3d_5/2_ core-level spectra can be divided into three symmetric components (Figure 6.), which can be assigned to Pd^0^ at 335.3–335.6 eV, Pd^2+^ at 336.5–336.6 eV, and Pd^4+^ at 337.8–338.0 eV.^47–50^ Interestingly, in contrast to copper, palladium reveals a majority of reduced speciation for (Pd/Cu)­TiO_2_ photocatalysts in comparison with those on the C_3_N_4_ substrate. Pd metal in the zero-valent state (Pd^0^) represents 75% and 51% of all palladium atoms in (Pd/Cu)^0.5%^TiO_2_ (Figure 6a) and (Pd/Cu)^1%^TiO_2_ samples (Figure 6c), respectively, and only approximately 17% for both photocatalysts on C_3_N_4_ (Figure 6b,d). On the other hand, the Pd^4+^ oxidation state is the dominant speciation for the samples on carbon nitride, i.e., 68% and 69% for (Pd/Cu)^0.5%^C_3_N_4_ and (Pd/Cu)^1%^C_3_N_4_, respectively, whereas the concentration of palladium ions bonded in PdO_2_ is only 15% for 0.5% (Pd/Cu)^0.5%^TiO_2_ and 37% for (Pd/Cu)^1%^TiO_2_.

**Figure 6:**
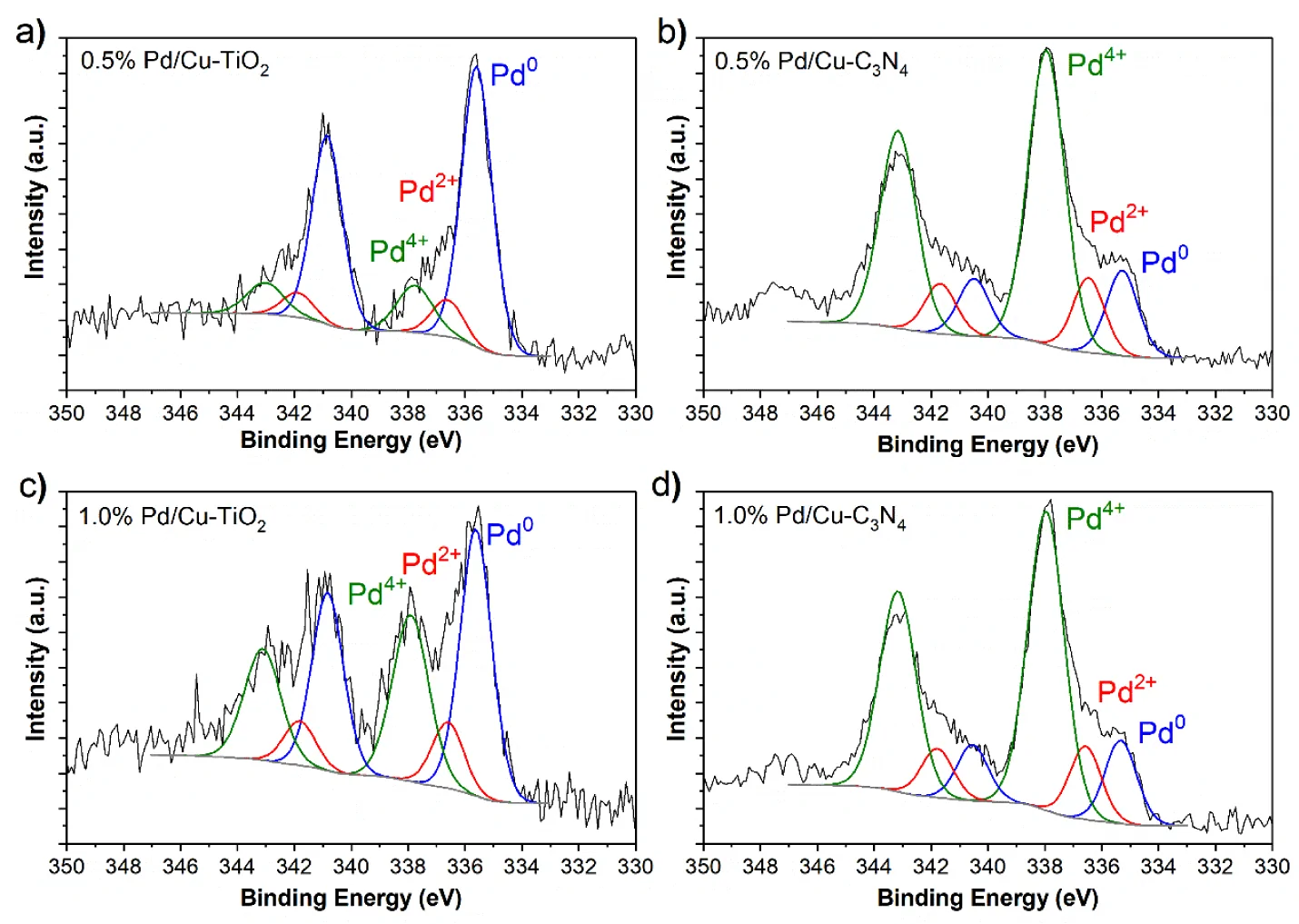
Palladium Pd 3d XPS spectra of a, c) (Pd/Cu)­TiO_2_ and b, d) (Pd/Cu)­C_3_N_4_ photocatalysts.

The textural properties of the synthesized materials were evaluated using N_2_ adsorption–desorption isotherms (Figure S3). The adsorption–desorption isotherms for both materials exhibit classic IUPAC Type IV profiles with well-defined hysteresis loops at higher relative pressures. This shape indicates the presence of prominent mesoporous frameworks and interstitial voids within the nanoparticle aggregates, which matches with the morphology observed via SEM and HAADF-STEM imaging. As expected for both TiO_2_ and C_3_N_4_ supports, the incorporation of bimetallic Pd/Cu nanoparticles led to a decrease in the Brunauer–Emmett–Teller (BET)-specific surface area (Table S2). The BET surface area of pristine TiO_2_ dropped from 10.5 m^2^/g to 5.6 m^2^/g for the (Pd/Cu)^1%^TiO_2_ sample. Interestingly, the calculated pore size distributions, derived via the BJH (Barrett–Joyner–Halenda) method from the nitrogen desorption isotherms, showed an apparent shift toward larger diameters (from 20.4 to 49.8 nm) following bimetallic loading. A similar trend of decreasing surface area and increasing average pore size was observed for the C_3_N_4_-based photocatalysts at higher metal loadings. Rather than indicating a structural expansion of the semiconductor support, this shift is attributed to a selective pore-blocking mechanism. The photodeposited Pd/Cu nanoparticles preferentially nucleate within or physically block the smaller mesopores of the pristine support. Consequently, these narrow channels become inaccessible to nitrogen gas, which artificially shifts the mathematical average of the remaining accessible pore volume toward larger, interparticle macroporous voids. The observed decrease in specific surface area accompanied by an increase in average pore diameter is well known in the literature for supported metal nanoparticles.^51,52^ It indicates that during the photodeposition process, the bimetallic Pd/Cu nanoparticles preferentially nucleate within or physically block the smaller mesopores of the support. Consequently, these smaller pores become inaccessible to nitrogen gas during the physisorption measurement, which reduces the total specific surface area and artificially shifts the mathematical average of the remaining accessible pore size distribution toward larger, interparticle macropores.^53^

Crucially, the long-term structural stability and metal-leaching resistance of these specific Pd/Cu bimetallic formulations have been rigorously validated in our previous work.^24^ Multicycle reusability tests demonstrated that the catalysts retain their high crystalline integrity and photocatalytic activity over four consecutive cycles. Furthermore, inductively coupled plasma-optical emission spectroscopy (ICP-OES) analysis of the treated effluent confirmed that leaching of Cu and Pd ions remained below the limit of detection (LOD), demonstrating the exceptional thermodynamic stability of the photodeposited alloy structure and confirming its safety for environmental discharge.

### Catalytic Activity

In this study, we examined how bimetallic TiO_2_- and C_3_N_4_-based nanoscale catalysts influence photocatalytic ozonation treatment. Titanium dioxide (TiO_2_) in the rutile form and graphitic carbon nitride (C_3_N_4_) have been chosen as investigators since TiO_2_ has benefits such as low solubility and high-surface area^54^ and the appropriate band gap of C_3_N_4_ is 2.7 eV that can be easily modified for achieving high photocatalytic efficiency.^55,56^ The materials can currently comprise a variety of compositions, morphologies, and topologies (spheres, nanotubes, nanowires, nanorods, powders, thin films).^57^ It is well known that morphology is important for microscale materials, since it is related to the proficiency of contaminants removal, and nanoscale materials are highly effective due to their multidimensional properties, such as small size and diverse morphology.^58,59^ In the catalyst ozonation process, nanoscale catalysts outperform microscale ones in catalytic activity and reaction efficiency due to their high surface-to-volume ratios and abundant, evenly distributed active sites. These features enhance ozone adsorption and activation, enable rapid mass transfer, and promote efficient ozone decomposition, resulting in faster reaction kinetics and superior practical performance.^60^ Therefore, the nanoscale bimetallic TiO_2_ and C_3_N_4_ catalysts have been employed for SMX and AMR degradation. One of the most important benefits of using these metal dopants is that they narrow the band gap between VB and CB, converting low-efficiency light absorbers into highly efficient UV–visible light absorbers,^61,62^ and eventually generating highly oxidizing species.^63^ Cu nanoparticles exhibit localized surface plasmon resonance (LSPR) in the visible light range, typically around 580 nm, due to collective oscillations of their conduction electrons when excited by visible light.^64^ Pd nanoparticles, on the other hand, display LSPR primarily in the UV region due to the high bulk plasma frequency of Pd.^65^ The bimetallic Pd/Cu catalysts enhanced LSPR properties leading to better catalytic performance.^66^ Moreover, the work function of Pd (∼5.6 eV) is higher than that of Cu (∼5 eV), the addition of Pd NPs increases the work function, and photoexcitation improves the transfer of electrons at the interface of catalysts.^66^ Taking this into account, in this study a sunlight simulator at 1000 W was applied to imitate visible light. Furthermore, apart from TiO_2_-rutile and C_3_N_4_-pure, modified catalysts with different Cu and Pd loadings (1 wt %, 0.5 wt %, 0.1 wt %) were also explored.

SMX degradation experiments, including adsorption, ozonation, photocatalytic oxidation, and photocatalytic ozonation over (Pd/Cu)­TiO_2_ and (Pd/Cu)­C_3_N_4_ were carried out to assess the efficacy of the catalytic activity. Under the present experimental conditions, SMX (100 μg/L) was minimally adsorbed by (Pd/Cu)^4%^C_3_N_4_ and (Pd/Cu)^6%^TiO_2_ after 30 min. After that time, the proper experiments were performed. Ozonation alone led to 50% SMX degradation within 30 min, while the photocatalytic ozonation significantly enhanced degradation causing full degradation after 5 min of treatment. The photocatalytic oxidation was not effective since full degradation was achieved only after 2 h. As depicted in Figure 7, both (Pd/Cu)­TiO_2_ and (Pd/Cu)­C_3_N_4_ catalysts exhibited higher first-order rate constants for SMX removal compared to ozonation alone, indicating an enhanced photocatalytic activity. Specifically, the (Pd/Cu)­C_3_N_4_ catalyst demonstrated a notably higher first-order rate constant than (Pd/Cu)­TiO_2_. Only slightly better oxidation was found for (Pd/Cu)­TiO_2_ compared to (Pd/Cu)­C_3_N_4_ when (Pd/Cu)^0.1%^ loading is considered. Pseudo-first-order rate constants for (Pd/Cu)­C_3_N_4_ and (Pd/Cu)­TiO_2_ were 2.7 and 2.4 times higher (at 1% loading), 1.8 and 1.6 times higher (at 0.5% loading), and 1.1 and 1.3 times higher (at 0.1% loading), respectively, compared to ozonation alone. As can be seen from Figure 7b, the photocatalytic oxidation of SMX led to first-order rate constants that were more than one order of magnitude lower.

**Figure 7:**
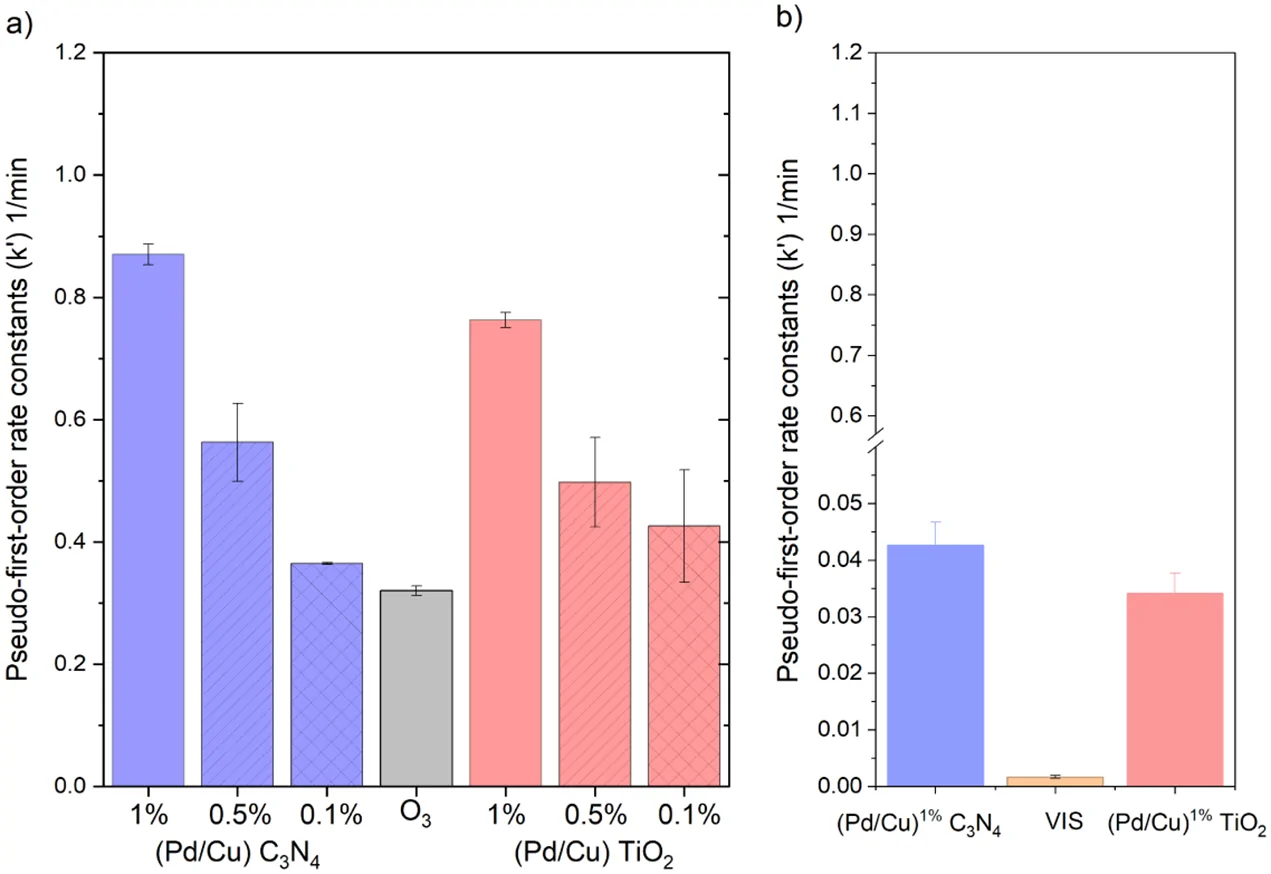
(a) The pseudo-first-order rate constants, k′ (1/min), during SMX photocatalytic ozonation in MQ water for different noble metal loads of C_3_N_4_- and TiO_2_-based catalysts compared to ozonation, and b) k′ for photocatalytic oxidation in MQ water.

To systematically isolate the individual contributions of photolysis, noncatalytic ozonation, and pristine semiconductor supports from the cooperative effects of the bimetallic cocatalysts, a consolidated operational and deactivation matrix was compiled (Table S1). Standalone photolytic controls (UV–visible light only) and noncatalytic ozonation (O_3_ only) runs established baseline log removal values (LRVs) for both taxonomic indicators and target antibiotic resistance genes (ARGs) under identical hydrodynamic conditions. Comparing these baseline controls with the pristine semiconductor supports (TiO_2_ and C_3_N_4_) and their mono- and bimetallic modifications reveals that the dramatic acceleration in genomic degradation is not merely an additive effect of bulk oxidant reactions but is driven by a genuine bimetallic (Pd/Cu) cooperative synergy that enhances charge-carrier separation and localized reactive species generation. In terms of metal loading, Figure 8a,b indicate that the best loading of bimetallic nanoparticles in terms of targeted gene degradation (namely, 16S rDNA, *bla*_TEM_, 23S rRNA, *intl*1) by photocatalytic ozonation is found in (Pd/Cu)^0.5%^C_3_N_4_ and (Pd/Cu)^0.1%^TiO_2_, whereas the best loading for the degradation of *intl*1 and 23S are found in TiO_2_-rutile and pure C_3_N_4_, respectively. Additionally, when the photocatalytic oxidation treatment processes were investigated, (Pd/Cu)^0.1%^TiO_2_ indicated the best performance for the degradation of targeted genes (16S rDNA, 23S rRNA, and *intl*1); however, the gene of *bla*_TEM_ is more sensitive to TiO_2_-rutile (Figure 8c). In contrast to TiO_2_-based catalysts, C_3_N_4_-pure and bimetallic (Pd/Cu)­C_3_N_4_ did not reveal significantly different removal efficiencies for the genes (16S rDNA, 23S rRNA, and *bla*_TEM_); only (Pd/Cu)^0.1%^C_3_N_4_ showed substantially reduced achievement on the *intl*1 gene (Figure 8d).

**Figure 8:**
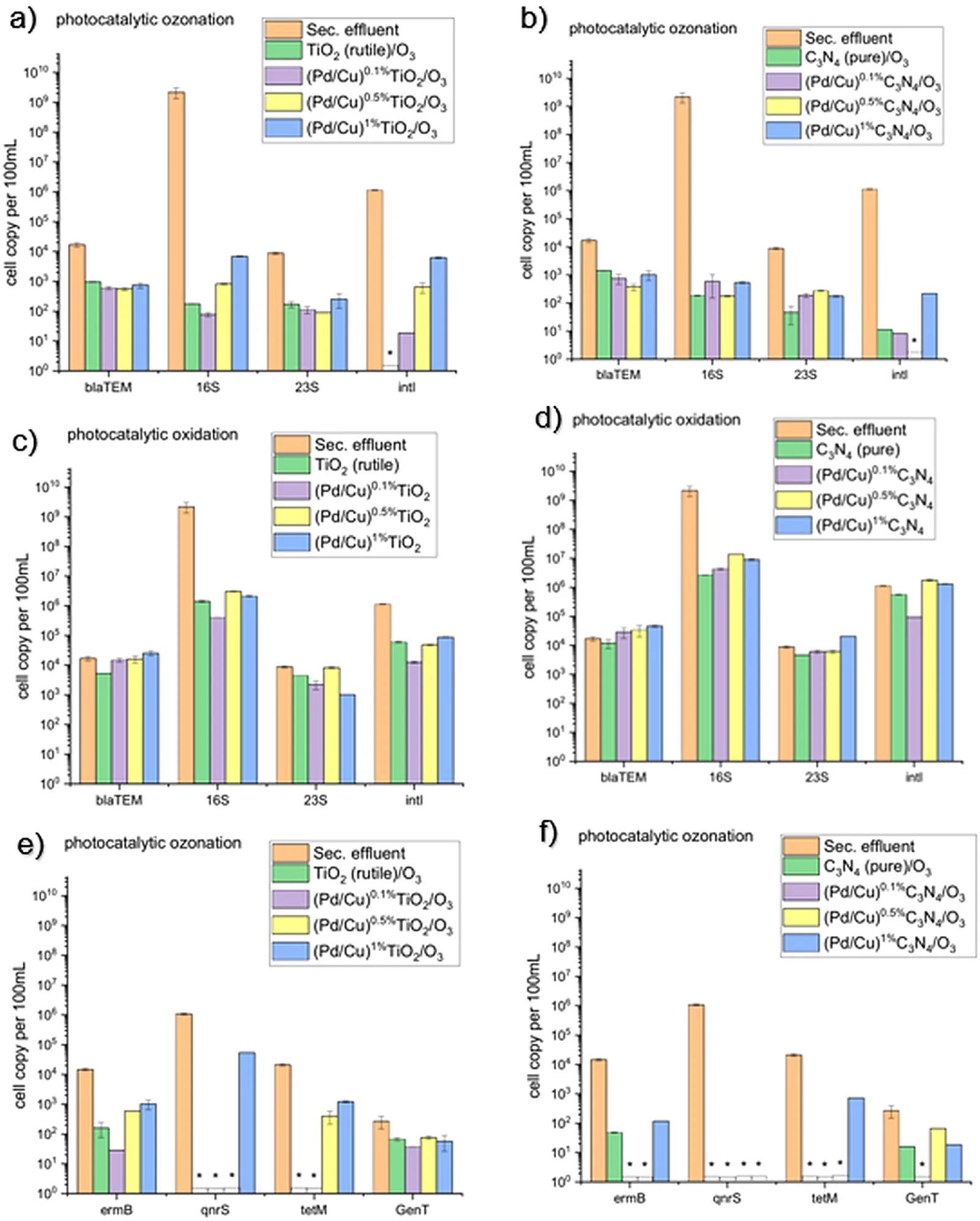
Comparison of applied catalysts’ efficiency in removing ARGs. Photocatalytic ozonation of *bla*_TEM_, 16S, 23S, *intl*1 on a) TiO_2_-based catalyst and b) C_3_N_4_-based catalyst. Photocatalytic oxidation of *bla*_TEM_, 16S, 23S, *intl*1 on c) TiO_2_-based catalyst and d) C_3_N_4_-based catalyst. Photocatalytic ozonation of *erm*B, *qnr*S, *tet*M, *Gen*T on e) TiO_2_-based catalyst and f) C_3_N_4_-based catalyst. *Blank column: below quantification/detection limit.

The observed hierarchy of genetic resistance, where the eubacterial housekeeping gene (16S rDNA) is readily inactivated while the antibiotic resistance gene *bla*_TEM_ exhibits extreme robustness, is highly consistent with our previous findings under solar AOP configurations.^24,27^ This structural and kinetic resilience is largely dictated by two factors: the intracellular vs extracellular state of the genetic material, and the specific nucleotide composition of the host bacteria. As previously demonstrated, *bla*_TEM_ genes are often located on highly robust plasmid vectors that protect them from immediate radical degradation, requiring prolonged exposure to achieve complete genomic cleavage.^67^ Conversely, taxonomic markers such as 23S rDNA (*Enterococci*) are rapidly eliminated under combined catalytic ozonation. This is due to the low guanine-cytosine (GC) content (<40%) of the *Enterococcus* genome, which significantly reduces the thermodynamic stability of its double-stranded DNA compared to GC-rich strains, rendering its genomic machinery highly susceptible to single-strand and double-strand breaks induced by surface-bound reactive species.^68^ To further contextualize these bimetallic semiconductor junctions within the latest state of the art, recent advancements in bimetallic catalyst engineering were considered. It was demonstrated that bimetallic loading on structured semiconductors drastically alters the charge–carrier distribution and surface adsorption properties and provides a dense network of active sites that accelerates the generation of reactive oxygen species (ROS).^69–71^

These findings clearly indicate that the mechanisms of action of both bimetallic catalysts differ as well as that despite those differences, susceptibility to that mechanism is dependent on the target. This is clearly visible during photocatalytic ozonation when inactivation of *erm*B, *qnr*S, *tet*M, and *Gen*T was investigated (Figure 8e,f). In general, although in the photocatalytic ozonation process, (Pd/Cu)^0.5%^C_3_N_4_ is depicted as the best loading for ARG removal (based on Figure 8b), the results observed for the 0.5 and 0.1 wt % (Pd/Cu)­C_3_N_4_ catalysts did not differ significantly in terms of *qnr*S, *erm*B and *tet*M (both catalysts caused complete inactivation). Hence, complete *Gen*T inactivation was observed for (Pd/Cu)^0.1%^C_3_N_4_. However, (Pd/Cu)^0.1%^TiO_2_ was observed to have the best performance in removing ARGs (*erm*B, *qnr*S, *tet*M, and *Gen*T).

Several trials have revealed that higher loadings of metallic nanoparticles result in a higher rate of contamination elimination.^72,73^ Moreover, Soares et al., 2011, has reported that the 1% Pd–1% Cu (wt %) bimetallic catalyst was one of the most potential candidate in nitrate reduction.^74^ Furthermore, according to,^75^ TiO_2_-based catalysts with the least amount of metal may diminish the scavenging impact of photogenerated electrons, resulting in the reoccurrence of the recombination phenomenon. However, the outcomes of our investigation do not support the predicted hypothesis. Although 0.1 wt %-doped catalysts indicated the best performance, the dependence was different for SMX removal in MQ water. Moreover, according to the results of the regrowth research, the 1.0 wt % Cu/Pd-doped catalysts exhibited the highest inhibitory performance on post-treatment bacterial regeneration (Figure 9), prompting their selection for the majority of our experimental trials. Since the BET surface area is noticeably reduced after Pd/Cu modification, the enhanced efficiency of the bimetallic samples toward taxonomic and ARG deactivation cannot be ascribed to an increase in physical adsorption sites. Instead, this behavior highlights a critical operational and biological design paradox. Lower metal loadings (0.1 and 0.5 wt %) provide a highly active, highly dispersed distribution of ultrasmall cocatalyst nanoparticles on the support surface, optimizing initial electron–hole pair separation and localized surface plasmon resonance (LSPR) effects.^73^ However, while these dispersed sites excel at the rapid primary chemical degradation of dissolved compounds, they generate a milder, highly diffuse oxidative environment that inflicts only sublethal cellular and membrane injuries on robust bacterial populations. During subsequent post-treatment dark storage, surviving bacteria are capable of repairing their damaged membranes and resuscitating. To achieve complete, sustained disinfection and prevent post-treatment regrowth, a higher density of active metal cocatalyst sites (1.0 wt %) is strictly indispensable. The localized concentration of Pd/Cu cocatalysts at 1.0 wt % drives a rapid, massive burst of highly localized reactive species that inflicts irreversible lipid peroxidation and complete physical lysis of the bacterial cell wall, thereby permanently destroying the cellular machinery required for DNA repair.

**Figure 9:**
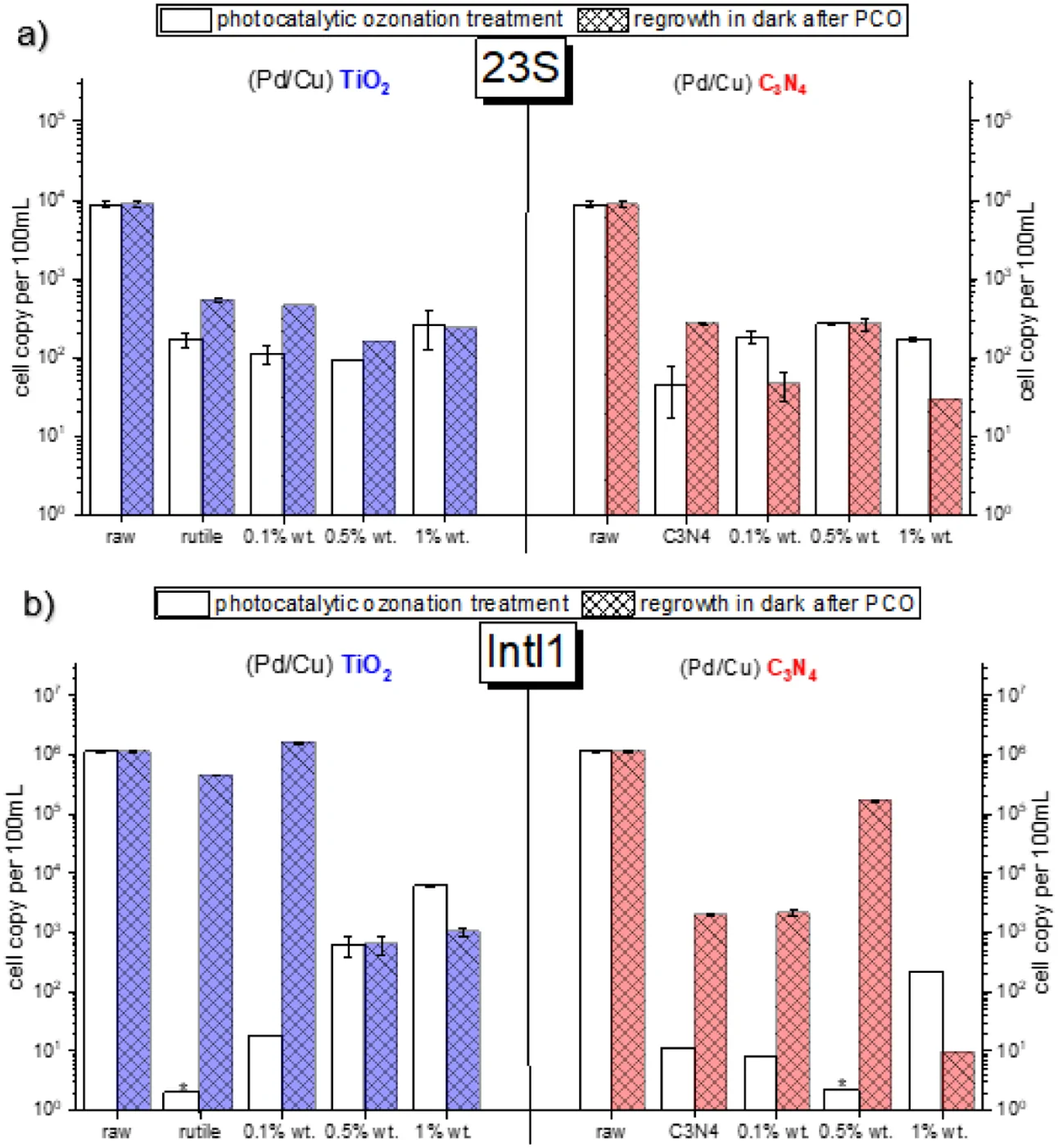
Assessing the effectiveness of both catalysts with diverse metallic dopants in inhibiting bacterial regrowth and eliminating genes 23S rRNA and *intl*1. a) Removal of 23S gene on left - (Pd/Cu)­TiO_2_ catalyst, right - (Pd/Cu)­C_3_N_4_ catalyst b) removal of *intl*1 gene on left - (Pd/Cu)­TiO_2_ catalyst, right - (Pd/Cu)­C_3_N_4_ catalyst.

As previously stated, ozonation could be effective in removing ARB and ABGs living in municipal wastewater effluents; however, from a practical and cost-effective standpoint, an excessive dose of ozone appears unnecessary due to the presence of ozone-resistant microorganisms that are hardly removed even when the ozone dose is increased. The purpose of this part is to verify the influence of Cu/Pd at various loadings (0.1, 0.5, and 1 wt %) on the degradation of pollutants. The transferred ozone dose (TOD) was measured over time to compare the processes in terms of ozone consumption. The greater the TOD value, the more ozone was transferred from the gas to the liquid phase, resulting in more ROS production and consumption. Moreover, at the same applied ozone dose, several AOP treatment scenarios with the greatest TOD values also appear to have the largest mass transfer yield. Treatment rates, initial ozone concentration, pH, water matrix, and other parameters all influence transfer yield.^76^ To put it another way, the ozone mass transfer yield was calculated as the ratio of transferred to injected ozone doses.

When the ozone profiles of single ozonation and photocatalytic ozonation are compared (Figure 10), it is apparent that the ozone transfer yield of ozonation is substantially lower than that of photocatalytic ozonation, since light accelerates ozone depletion and optimizes the transfer yield.^77^ Photocatalytic ozonation is more efficient in removing ARGs than ozonation, which supports the behavior seen in relation to ozone consumption. When the catalyst’s influence on ozone mass transfer is considered, photocatalytic ozonation with (Pd/Cu)^0.5%^C_3_N_4_ has the best performance in enhancing ozone transfer from gas to liquid, as shown in Figure 10b,c), which could be explained by the acceleration of ozone consumption by a variety of ROS. The conclusions of this section also demonstrate that (Pd/Cu)^0.5%^C_3_N_4_ is the most efficient in removing the ARGs. In contrast, in photocatalytic ozonation with (Pd/Cu)­TiO_2_-based processes, ozone consumption is accelerated to a much lesser extent compared to (Pd/Cu)­C_3_N_4_ photocatalytic ozonation (Figure 10b,c,e,f). (Pd/Cu)^0.1%^TiO_2_ exhibited the greatest performance in enhancing ozone transfer, as shown in Figure 10e,f, which also is in agreement with its catalytic activity toward ARGs.

**Figure 10:**
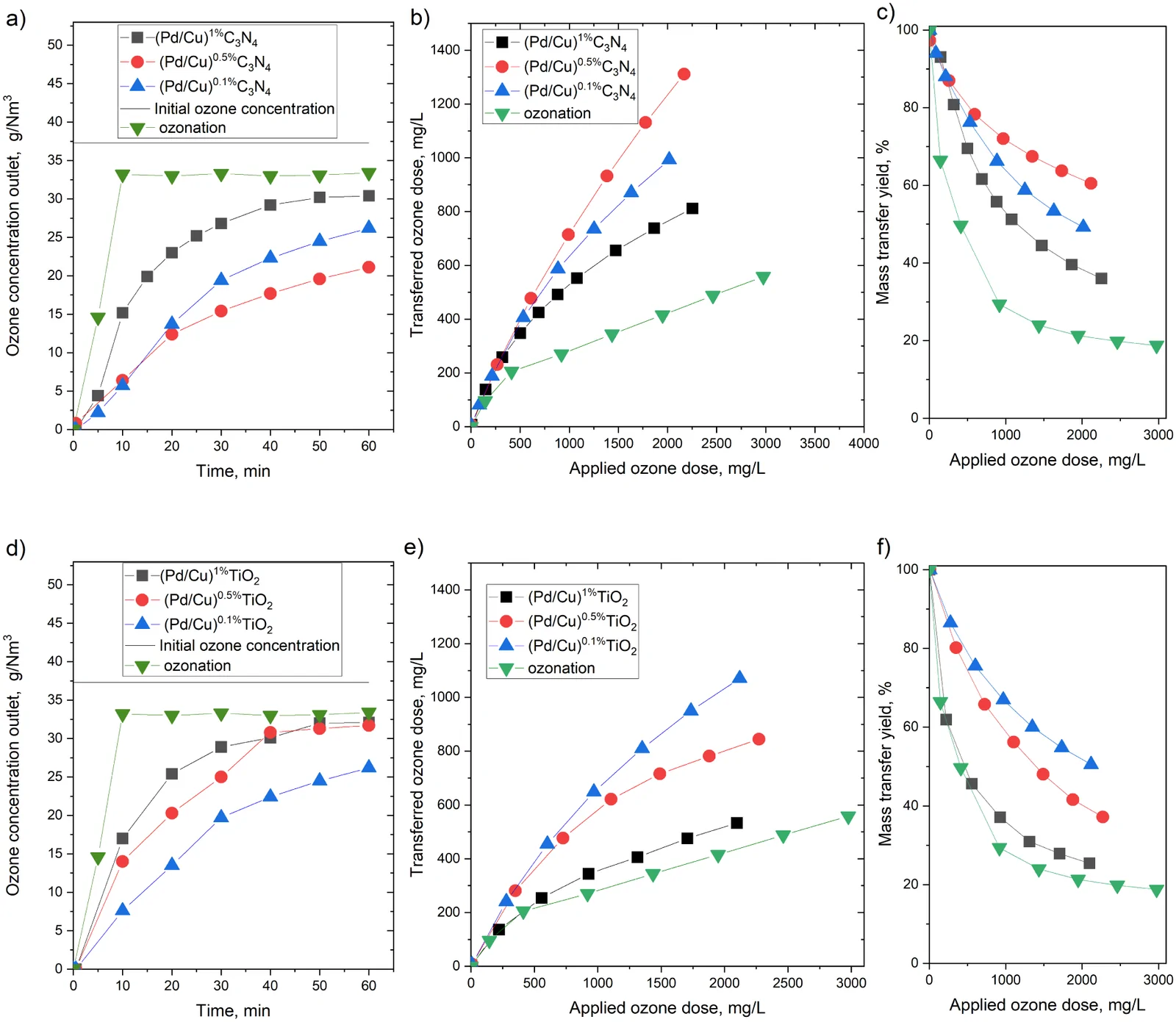
Inlet and outlet ozone concentration over time for a) (Pd/Cu)­C_3_N_4_ and d) (Pd/Cu)­TiO_2_; transferred ozone dose as a function of applied ozone dose for b) (Pd/Cu)­C_3_N_4_ and e) (Pd/Cu)­TiO_2_; ozone mass transfer yield along single ozonation and different modified catalyst photocatalytic ozonation treatment scenarios for c) (Pd/Cu)­C_3_N_4_ and f) (Pd/Cu)­TiO_2_ (gas inflow rate: 20 L/h; inlet ozone concentration: 37 mg L^–1^).

### Mechanism Investigation

In this study, we sought to unravel the intricate mechanisms and reaction pathways underlying Pd/Cu bimetallic TiO_2_- and C_3_N_4_-based photocatalytic processes. To investigate the role of ROS in photocatalytic degradation, as a first step the photocatalytic oxidation of SMX using (Pd/Cu)^1%^TiO_2_ and (Pd/Cu)^1%^C_3_N_4_ was carried out in the presence of ROS scavengers. The trapping experiments were carried out by using different scavengers to detect the involved species. It is well known that *t*-BuOH reacts with OH radicals (7.6 × 10^8^ M^–1^ s^–1^),^78^ therefore it was selected to capture ^•^OH. Ammonium oxalate (AO) was used as a hole scavenger (h^+^) under visible light.^79^ Sodium azide (NaN_3_) was selected due to its high reactivity toward singlet oxygen. However, it should be noted that NaN_3_ is efficient in singlet oxygen quenching (2 × 10^9^ M^–1^ s^–1^) but also may quench effectively hydroxyl radicals (1 × 10^9^ M^–1^ s^–1^).^80^ 1,4-benzoquinone (p-BQ) was applied as a superoxide radical ion (O_2_^•–^, 1.1 × 10^9^ M^–1^ s^–1^).^81^ However, p-BQ can also react with ^●^OH with the rate constant 1.2 × 10^9^ M^–1^ s^–1^.^82^ Additionally, the reaction was carried out in the presence of superoxide dismutase (SOD) and catalase as O_2_^•–^ and H_2_O_2_ scavengers, respectively.^83^

As can be seen in Figure 11, the addition of *t*-BuOH provokes a significant reduction of the degradation efficiency proving the involvement of the ^●^OH radicals in the process for both catalysts. However, the process was inhibited by ∼52% when (Pd/Cu)^1%^TiO_2_ was applied, while in the case of (Pd/Cu)^1%^C_3_N_4_, the inhibition was only 37%. When considering the reaction with p-BQ, there is evidence that in (Pd/Cu)^1%^TiO_2_ only a small influence is related to O_2_^●–^. When (Pd/Cu)^1%^C_3_N_4_ is employed, the oxidation mechanism is dominated by the superoxide radical anion. Due to the insufficient valence band potential of C_3_N_4_, direct water oxidation to hydroxyl radicals is thermodynamically unfavorable. Nevertheless, O_2_^●–^ can participate directly in oxidation reactions, act as a precursor for ^1^O_2_ formation, and contribute indirectly to trace hydroxyl radical generation via localized metal-mediated pathways. These include in situ H_2_O_2_ formation followed by Cu­(I)-driven Fenton-like reactions occurring at Pd/Cu surface sites. The results confirm that H_2_O_2_ is also present in the system during both photocatalytic processes. However, its presence is strongly related to the O_2_^●–^. Moreover, strong inhibition of the efficiency can be observed by NaN_3_. Taking into account that sodium azide interacts with both OH radicals and ^1^O_2_, it was found that SMX degradation in the presence of NaN_3_ slightly differs from that with *t*-BuOH and p-BQ for (Pd/Cu) ^1%^TiO_2_, indicating a low contribution of ^1^O_2_ to SMX degradation. However, a significant difference between the reaction in the presence of NaN_3_ compared to *t*-BuOH and p-BQ can be found for (Pd/Cu)^1%^C_3_N_4_ photocatalytic degradation, leading to remarkable contribution of singlet oxygen. The experiment proved also direct or indirect holes (by oxidizing surface OH groups and water producing OH radicals) in SMX decomposition for both catalysts. Furthermore, the confirmed presence of O_2_^●–^ and h^+^ can lead to ^1^O_2_ generation,^24^ especially when (Pd/Cu)^1%^C_3_N_4_ is used.

**Figure 11:**
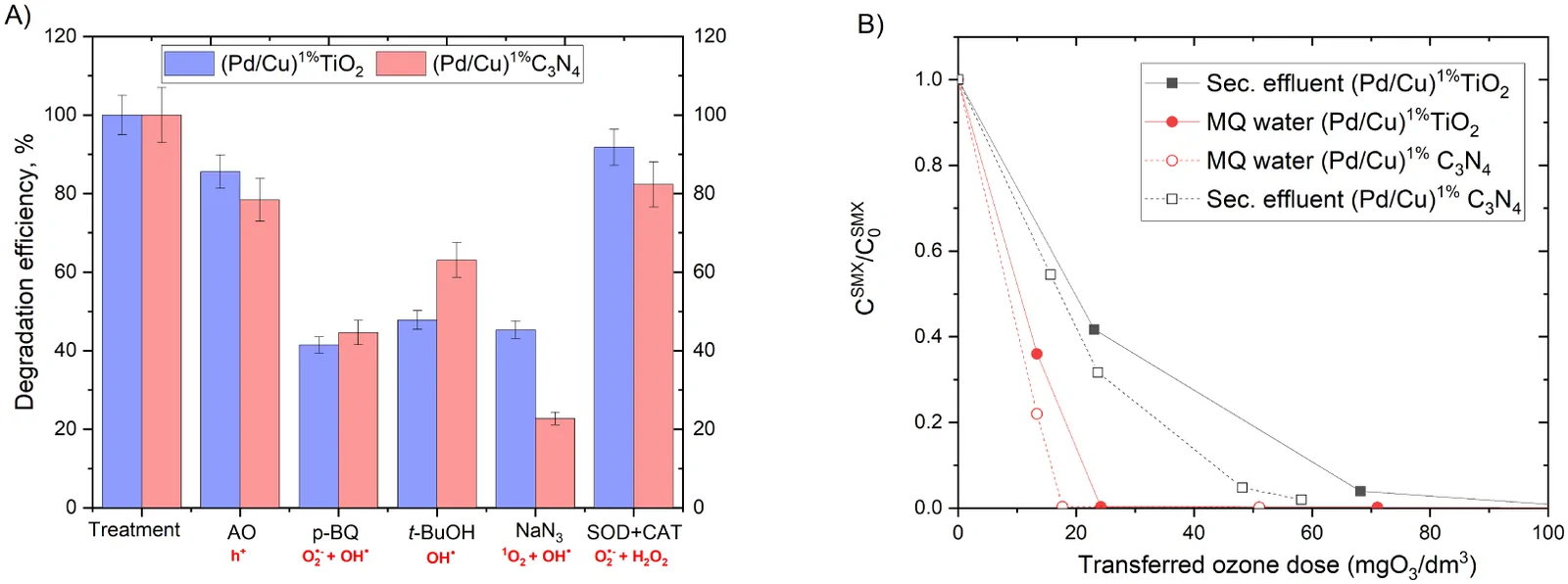
(a) Photocatalytic oxidation in Milli-Q water of SMX with bimetallic catalysts in the absence and presence of ROS scavengers under visible light; b) normalized concentration of SMX as a function of TOD during photocatalytic ozonation in different water matrices.

The photocatalytic ozonation of SMX has been investigated from the water matrix point of view. As can be seen in Figure 11b, for both investigated catalysts (Pd/Cu)^1%^TiO_2_ and (Pd/Cu)^1%^C_3_N_4_, better performance has been observed in pure water than in secondary effluent. A higher transferred ozone dose is required in secondary effluents to achieve complete SMX degradation. In MQ water, photocatalytic ozonation with (Pd/Cu)^1%^C_3_N_4_ and with (Pd/Cu)^1%^TiO_2_ caused complete SMX removal when TOD reached 17.7 mgO_3_/dm^3^ and 24.2 mgO_3_/dm^3^, respectively. In secondary effluents, a TOD equal to 58.2 mgO_3_/dm^3^ led to 98% SMX degradation when (Pd/Cu)^1%^C_3_N_4_ was used, while 68.2 mgO_3_/dm^3^ was required to degrade 96% of SMX. The decrease in SMX efficacy and the increase in TOD are related to effluent dissolved organic matter (EfDOM), which is in agreement with refs 84,85 . In order to obtain information about the EfDOM fraction interaction during ozone-based processes, size exclusion chromatography (SEC) with organic carbon detection (OCD) was used. The LC-OCD chromatograms of untreated secondary effluent and after ozonation (O_3_), photolytic ozonation (O_3_/VIS), and during photocatalytic ozonation with (Pd/Cu)^1%^TiO_2_ and (Pd/Cu)^1%^C_3_N_4_ are presented in Figure S4. The untreated secondary effluents exhibit peaks corresponding to biopolymers, humic acids and building blocks, a small amount of low-molecular-weight acids, especially neutrals, and amphiphiles. Following ozone-based processes for all applied ones, the biopolymers were degraded, indicating that long-chain substances were degraded into lower-molecular-weight compounds with shorter chain lengths, which is in agreement with refs 86–88 . It can be observed that the fractions of HA and building blocks are present in higher amounts after photocatalytic ozonation, leading to values lower than the initial LMW neutrals value. This can suggest that biopolymers are transformed into HA/BB while LMW are going to be mineralized during that process. The higher HA content during photocatalytic ozonation can be related to lower susceptibility toward ^●^OH radicals compared to O_3_.^89^ Nevertheless, the obtained results clearly showed that EfDOM influences ozone-based processes due to competition for oxidants. Considering that ozone-based technologies interact with EfDOM^90^ which has been confirmed by our study, the mechanism of photocatalytic ozonation in secondary effluent was investigated from the ozone consumption point of view. As can be seen in Figure S5, the ozone outlet concentrations under different systems are presented. The results indicated that ozonation in secondary effluents proceeds initially with much higher ozone consumption; hence, photolytic ozonation also accelerates ozone consumption. Moreover, the addition of the catalysts significantly decreased *O*_3_^G,out^ compared to O_3_ in WW. Among the catalytic ozonation systems, different profiles can be observed with and without doping of (Pd/Cu) for both catalysts, indicating that catalysts with (Pd/Cu) content have more active sites to decompose ozone.

As a second stage of the mechanism investigation, additional experiments were performed during photocatalytic ozonation of effluents. It should be noted that during catalytic ozonation the role of ROS is very complex.^83,91,92^ Therefore, the investigation in this case is mostly focused on the evaluation of ROS in ARG inactivation rather than precisely indicating which ROS is responsible for inactivation. However, based on the results from the SMX mechanism examination, the influence of some specific ROS which was proved can be considered. The influence of scavenger agents on 1%-doped bimetallic photocatalytic ozonation (TiO_2_, C_3_N_4_) was examined, with sodium azide and sodium sulfate being employed for investigation in the former case, while benzoquinone and *tt*-BuOH were utilized for the latter. Sulfite (Na_2_SO_3_) was applied as O_3_ scavenger (1.5 × 10^9^ M^–1^ s^–1^).^93^ It should be noted that Na_2_SO_3_ is also used as a H_2_O_2_ scavenger;^94^ however, the second-order rate constant for the oxidation of sulfite with hydrogen peroxide is much lower (6 × 10^3^ M^–1^ s^–1^ in acidic and 0.74 M^–1^ s^–1^ in alkaline solution.^95,96^ As mentioned, *t*-BuOH can be applied as an OH scavenger; however, in catalytic ozone-based processes, it can react slowly with ozone (3 × 10^–5^ M^–1^ s^–1^).^78^ Moreover, NaN_3_ reacts slowly with O_3_ (protonated form <4 × 10^3^ M^–1^ s^–1^, deprotonated 4 × 10^6^ M^–1^ s^–1^).^93^ Also, p-BQ can react with ozone (2.5 × 10^3^ M^–1^ s^–1^).^82^ However, it should be noted that scavengers such as *t*-BuOH, NaN_3_, or p-BQ must be carefully considered when interpreting catalytic ozonation mechanisms, as they not only quench selected ROS but also modify the kinetics of O_3_ decomposition and compete with EfDOM for access to the catalyst surface.^82,91,97,98^ Therefore, the interactions between the ROS and the selected scavenger need to be considered. The application of scavenging experiments for ROS mechanism involvement is commonly applied in ARG and ARB inactivation.^99–102^ As mentioned, the scavengers can influence O_3_ decomposition as in this regard the role of ROS can be overlooked or underestimated.^100^ Thus, the ozone consumption during scavenging experiments was investigated. A rigorous kinetic analysis based on film theory was applied to decouple chemical ozone consumption from physical mass transfer dynamics. The catalytic decomposition of dissolved ozone at the bimetallic catalyst surface creates a steeper local ozone concentration gradient at the gas–liquid boundary layer. This concentration gradient acts as a driving force, increasing the apparent volumetric mass transfer coefficient (*k_L_a*).^99^ However, our ozone-consumption profiles (Figure 12) reveal a critical methodological caveat: traditional radical scavengers (*t-*BuOH, NaN_3_, p-BQ) are not passive observers. Instead, they directly participate in the reaction by accelerating ozone decomposition and blocking the active catalytic sites of the bimetallic composites. Consequently, apparent increases in ozone consumption during scavenging assays must be interpreted as a combined effect of mass transfer enhancement and scavenger-induced ozone depletion, rather than purely representing bulk reactive oxygen species (ROS) production. This methodological challenge is highlighted when comparing our scavenger-consumption profiles with contemporary literature,^103^ which relies heavily on diagnostic scavengers and EPR spin-trapping. While EPR provides a snapshot of radical presence, it does not account for how these organic additives alter the physical mass transfer and decay kinetics of gaseous ozone. As shown in our work, the addition of *t-*BuOH or NaN_3_ drastically accelerates ozone consumption while physically competing for active catalyst surface sites. This creates a false impression of chemical scavenging of ROS in the bulk phase when, in reality, the physical interface and ozone transfer are being directly disrupted. Therefore, our model demonstrates that diagnostic scavenger assays in catalytic ozonation cannot be interpreted as simple, noninteractive chemical probes and must be validated alongside dynamic mass transfer coefficient profiling.

**Figure 12:**
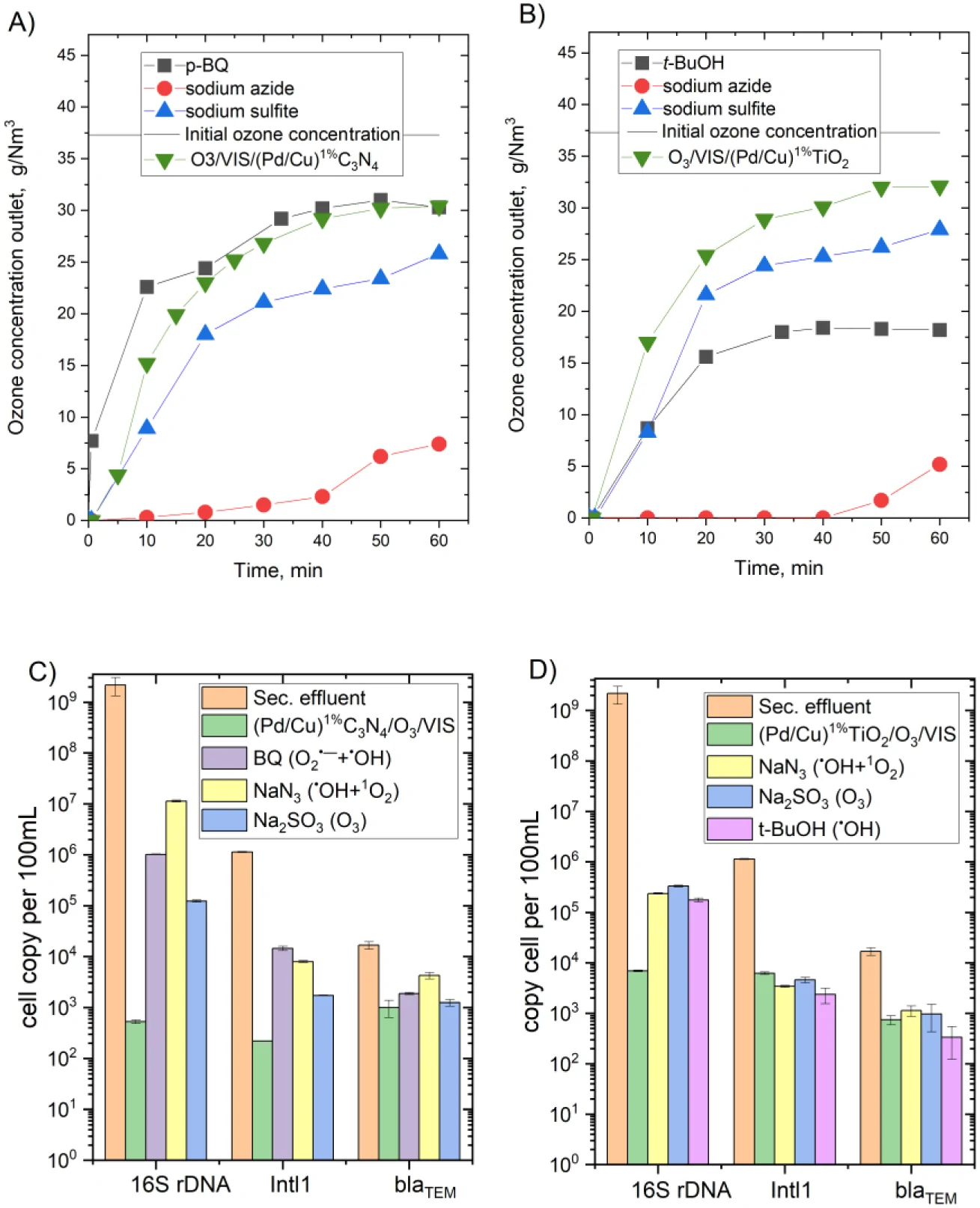
Inlet and outlet ozone concentration over time for a) (Pd/Cu)­C_3_N_4_ and b) (Pd/Cu)­TiO_2_ with selected scavengers. Comparison of the performance of c) (Pd/Cu)^1%^C_3_N_4_ and d) (Pd/Cu)^1%^TiO_2_ photocatalytic ozonation in removing 16S rDNA, *intl*1, and *bla*_TEM_ before and after adding scavengers.

As can be seen in Figure 12a,b, sodium sulfite increased ozone consumption during both catalytic processes, leading to the same inhibition of ROS mechanisms. Therefore, based on that finding, a similar influence on ARGs’ photocatalytic ozonation should be expected. As can be seen from Figure 12c,d, very similar inhibitory effects can be observed for 16S rDNA where the abundance of those ARGs was reduced by the same order of magnitude, indicating that O_3_ has the same impact in both processes. The abundance of *bla*_TEM_ has not changed during either catalytic process, suggesting that this pathway is not preferred. Aside from the fact that in the case of *intl*1 during (Pd/Cu)^1%^C_3_N_4_ photocatalytic ozonation some influence was observed, photocatalytic ozonation with (Pd/Cu)^1%^TiO_2_ in the presence of Na_2_SO_3_ did not influence the abundance.

The consumption of ozone in the presence of NaN_3_ behaved differently for both catalytic processes. As mentioned before, NaN_3_ can react with ^1^O_2_ and ^●^OH at a similar reaction rate, while it reacts very slowly with O_3_. The consumption of ozone during the reaction with NaN_3_ is therefore expected.^104^ However, the difference in the ozone profile suggests that during (Pd/Cu)^1%^C_3_N_4_ photocatalytic ozonation the consumption of NaN_3_ is related to ^1^O_2_ and ^●^OH, while in the case of (Pd/Cu)^1%^TiO_2_ those ROS are present in smaller amounts and are less responsible for the catalytic performance. This is clearly seen in Figure 12c,d, where a higher abundance of 16S rDNA, *bla*_TEM_ and *intl*1 is observed for the reaction with (Pd/Cu)^1%^C_3_N_4_, while only an influence on 16S rDNA can be observed when the process is carried out with (Pd/Cu)^1%^TiO_2_. The experiments with *t*-BuOH were performed only with (Pd/Cu)^1%^TiO_2_ photocatalytic ozonation, leading to increased ozone consumption; however, the only visible effect on 16S rDNA can be seen during that experiment. Surprisingly, the scavenging experiment with p-BQ during (Pd/Cu)^1%^C_3_N_4_ photocatalytic ozonation caused a slight inhibition in ozone consumption. It can be related to blocking the active sites of the catalysts,^91^ or it can indicate that ozone is not taking part in the scavenging reaction. The influence of p-BQ on 16S rDNA, *bla*_TEM_, and *intl*1 is visible and can also be related to O_2_^●–^ and ^●^OH.

Notably, when focusing on TiO_2_-based photocatalytic ozonation, the scavengers had limited influence on the elimination of *intl*1, indicating that ROS such as hydroxyl radicals, singlet oxygen, and ozone might not be the primary drivers of *intl*1 deactivation; however, in the case of 16S rDNA, the ROS influence is noticeable. Considering *intl*1, it is plausible that alternative mechanisms involving the participation of holes, electrons, or other mechanical pathways should be considered. This can be supported by XPS analysis, where palladium existed primarily in a metallic, zero-valent state, acting as an electron sink to facilitate direct charge transfer. Therefore, this suggests a mechanism driven by direct hole or electron transfer rather than a bulk radical attack.

The proposed divergence in the reaction pathway, where C_3_N_4_ operates via a dominant bulk-phase ROS mechanism (^1^O_2_ and O_2_^•–^) while TiO_2_ relies on a direct surface-bound charge-transfer pathway, is strongly supported by both our previous photoluminescence data^24^ and current XPS observations. In our previous work, the PL spectra of bimetallic TiO_2_–CuPd showed a near-complete quenching of excitonic recombination, proving that photodeposited Pd acts as a powerful Schottky electron trap. This is chemically validated by our XPS results (Figure 5), which reveal that palladium exists predominantly in its zero-valent metallic state (Pd^0^, up to 75%) on the TiO_2_ support, compared to only 17% on the C_3_N_4_ support. On TiO_2_, the Pd^0^ nanoparticles act as highly efficient electron sinks, establishing a Schottky barrier at the metal–semiconductor interface that drives rapid charge separation. This spatial separation allows photogenerated holes (h^+^) to accumulate at the TiO_2_ surface, where they can directly oxidize adsorbed organic targets and deactivate recalcitrant genetic sequences like *intl*1 via direct charge transfer, bypassing the necessity of bulk radical intermediates and rendering the process remarkably resistant to conventional chemical scavengers. This model of surface-confined direct charge transfer aligns with state-of-the-art semiconductor heterojunction theories,^105^ which demonstrate that localized metallic junctions create a powerful internal electric field that guides photogenerated carriers directly to adsorbed substrates, minimizing energy-loss through bulk solution recombination.

Conversely, in the context of C_3_N_4_-based photocatalytic ozonation, the scavengers employed exhibited a considerable reduction in catalytic activity. Specifically, sodium azide and benzoquinone demonstrated pronounced impacts on the removal of the 16S rDNA gene and *intL1*, respectively. Thus, it can be inferred that superoxide ion radicals, ozone, and singlet oxygen collectively contribute to the elimination of ARGs, thereby elucidating the more efficient removal of ARGs observed with the C_3_N_4_ catalyst compared to that with TiO_2_. This implies that in C_3_N_4_-based treatment processes, ROS plays a dominant role in pollutant removal, whereas other factors may underlie the removal mechanisms mediated by TiO_2_-based AOPs. These findings can be also supported by XPS analysis, where in the case of C_3_N_4_-based catalysts palladium was predominantly oxidized (Pd^4+^), while copper existed largely as Cu^+^, creating a redox-active surface conducive to the generation of ROS.

Overall, our results underscore the notion that distinct AOP conditions may exhibit diverse treatment mechanisms, with scavenger agents proving to be instrumental in unveiling the major influences in photocatalytic ozonation. However, further research is warranted to ascertain the primary hydroxyl radicals involved in the treatment processes. Moreover, as can be seen, the 1 wt % Cu/Pd catalyst treatment approach performed badly in terms of removing ARGs, which is also reflected in the worst boosting of ozone transfer. Nevertheless, both 1 wt % Cu/Pd catalysts were effective in terms of preventing bacterial regrowth. However, regarding the efficiency toward *bla*_TEM_ including bacterial regrowth (Figure S6), the abundance for both catalytic ozonation processes was similar. This might be explained by the mechanism which states that because the two catalysts had distinct mechanisms of action, ROS did not play a significant role in the deactivation of the ARGs.

## Conclusions

This study successfully elucidated the distinct mechanisms and efficiencies of bimetallic Pd/Cu-modified TiO_2_ and C_3_N_4_ catalysts in the photocatalytic ozonation of sulfamethoxazole (SMX) and antibiotic-resistant genes (ARGs). Both bimetallic catalysts significantly enhanced degradation rates compared to those of ozonation alone, with (Pd/Cu)­C_3_N_4_ generally exhibiting higher pseudo-first-order rate constants than (Pd/Cu)­TiO_2_.

The investigation of reaction mechanisms using radical scavengers revealed fundamental differences between the two materials. For C_3_N_4_-based photocatalytic ozonation specifically, superoxide radicals, singlet oxygen, and ozone play a dominant role in pollutant elimination. Conversely, TiO_2_-based processes exhibited a different behavior, where ROS appeared less critical for the deactivation of specific targets like *intl*1, suggesting the involvement of alternative pathways such as direct hole or electron interactions.

Furthermore, the study highlighted a trade-off between the removal efficiency and regrowth inhibition. While lower metal loadings (0.1 and 0.5 wt %) demonstrated the highest performance for the initial removal of ARGs, the 1 wt % Pd/Cu loading was necessary to effectively prevent bacterial regrowth after treatment. Additionally, the efficiency of both systems was influenced by the water matrix; the presence of EfDOM in secondary effluents increased the required ozone dose by competing for oxidants and affecting the ozone decomposition profiles.

Ultimately, (Pd/Cu)^0.5%^C_3_N_4_ demonstrated the best overall performance in enhancing ozone mass transfer and removing ARGs driven by its capacity to accelerate ozone consumption through a variety of ROS. These findings underscore that while both catalysts are effective, their optimal application depends on the specific target pollutants and the necessity of controlling bacterial regrowth with C_3_N_4_ showing particular promise for ROS-driven remediation strategies.

## Supplementary Material



## Notes

Extra SEM and HAADF-STEM/EDXS images, BET adsorption–desorption isotherms, LC-OCD chromatograms, ozone uptake profiles, and bacterial regrowth data (DOCX)

## Notes

This work is dedicated to the memory of Prof. Dr. Thomas Schwartz, a great supervisor, mentor, and colleague.
