# Supplementary material for: Mechanistic Divergence and Ozone Mass Transfer Enhancement in Photocatalytic Ozonation: A Comparative Study of Bimetallic Pd/Cu-Modified Rutile TiO2 and g‑C3N4
Source: ACS Omega. 2026 Oct 13;11(42):65801–20. doi: 10.1021/acsomega.6c09130 (PMC13717668; doi:10.1021/acsomega.6c09130)
Supplement: Supplementary file 1 [file ao6c09130_si_001.docx]

**SUPPLEMENTARY DATA:**

Mechanistic Divergence and Ozone Mass Transfer Enhancement in Photocatalytic Ozonation: A Comparative Study of Bimetallic Pd/Cu Modified Rutile TiO_2_ and g-C_3_N_4_

Marta Gmurek^1,2,3,*^, Cong Xiaoyu^2^, Jacek Balcerzak^4^, Paweł Mazierski^5^, Magdalena Miodyńska-Melzer^5^, Tomasz Klimczuk^6^, Adriana Zaleska-Medynska^5^, Dominik Knozowski^1^, Radian Popescu^7^, Thomas Schwartz^2^, Harald Horn^3,8^

^1^Institute of General and Ecological Chemistry, Faculty of Chemistry, Lodz University of Technology, Zeromskiego 116, 90-924 Lodz, Poland

^2^Institute of Functional Interfaces (IFG), Karlsruhe Institute of Technology (KIT), Hermann von Helmholtz Platz 1, 76344 Eggenstein-Leopoldshafen, Germany

^3^Engler-Bunte-Institut, Water Chemistry and Water Technology, Karlsruhe Institute of Technology, Engler-Bunte-Ring 9a, 76131 Karlsruhe, Germany

^4^Division of Molecular Engineering, Faculty of Process & Environmental Engineering, Lodz University of Technology, Wolczanska 213, 93-005, Lodz, Poland

^5^Department of Environmental Technology, Faculty of Chemistry, University of Gdansk, Wita Stwosza 63, 80-308, Gdansk, Poland

^6^Department of Solid State Physics, Faculty of Applied Physics and Mathematics, Gdansk University of Technology, Narutowicza 11/12, 80-233, Gdansk, Poland

^7^Laboratory for Electron Microscopy, Karlsruhe Institute of Technology KIT, Finanzmanagement Kaiserstraße 12, D-76131 Karlsruhe, Germany

^8^DVGW German Technical and Scientific Association for Gas and Water Research Laboratories, Water Chemistry and Water Technology, Engler-Bunte-Ring 9a, 76131 Karlsruhe, Germany

*Corresponding author: [marta.gmurek@p.lodz.pl](mailto:marta.gmurek@p.lodz.pl)

**Table S1.** Consolidated control and combined treatment matrix for the inactivation of ARGs and taxonomic gene markers in secondary wastewater effluent. Operating Conditions: Reactor volume = 400 mL; Temperature = 10 °C; Catalyst dosage = 150 mg L^-1^ (where applied); Light source = Visible-light Xenon lamp (1000 W, 350–800 nm, photon flux 448 W m^-2^); Ozone gas inflow rate = 0.4 L min^-1^ at an inlet concentration of 10 mg L^-1^ (37 mg O_3_ Ndm^-3^). LOD = Limit of Detection. Based on presented results and our previously published study in Gmurek, M., et al. (2023) and Cong, X., et al. (2024).

| Process | Catalyst Support & Loading | Visible Light | Ozone Inflow | H2O2 (mM) | Time (min) | 16S rDNA Removal (LRV) | intl1 Removal (LRV) | blaTEM Removal (LRV) | 23S rDNA (Enterococci) (LRV) |
| --- | --- | --- | --- | --- | --- | --- | --- | --- | --- |
| Photolysis (Light alone) | None | Yes | No | 0 | 60 | <0.2 | <0.1 | 0.0 | <0.1 |
| Ozonation (O_3_ alone) | None | No | Yes | 0 | 60 | 4.5 | 1.1 | 3.2 | 1.0 |
| Photolytic Ozonation | None | Yes | Yes | 0 | 60 | 4.8 | 3.9 | 2.0 | 1.2 |
| Photocatalysis (Pristine TiO_2_) | Pristine Rutile TiO_2_ | Yes | No | 0 | 60 | 3.0 | 1.0 | 0.0 | 1.0 |
| Photocatalysis (g-C_3_N_4_) | Pristine g-C_3_N_4_ | Yes | No | 0 | 60 | 2.3 | 0.0 | 0.0 | 0.0 |
| Photocatalysis (Bimetallic TiO_2_) | (Pd/Cu)^1%^TiO_2_ | Yes | No | 0 | 60 | 3.5 | 1.2 | 0.0 | 1.1 |
| Photocatalysis (Bimetallic C_3_N_4_) | (Pd/Cu)^1%^g-C_3_N_4_ | Yes | No | 0 | 60 | 2.5 | 0.0 | 0.0 | 0.0 |
| Catalytic Ozonation (TiO_2_) | Pristine Rutile TiO_2_ | No | Yes | 0 | 60 | 3.8 | 1.3 | 0.4 | 1.1 |
| Catalytic Ozonation (C_3_N_4_) | Pristine g-C_3_N_4_ | No | Yes | 0 | 60 | 4.0 | 1.5 | 0.6 | 1.2 |
| Photocatalytic Ozonation (TiO_2_) | (Pd/Cu)^1%^TiO_2_ | Yes | Yes | 0 | 60 | 5.5 | 2.3 | 1.4 | 1.7 |
| Photocatalytic Ozonation (C_3_N_4_) | (Pd/Cu)^1%^g-C_3_N_4_ | Yes | Yes | 0 | 60 | 6.5 | 3.7 | 1.2 | 1.8 |
| Peroxone alone | None | No | Yes | 0.92 | 60 | 5.0 | 1.8 | 1.0 | 1.3 |
| Photocatalytic Peroxone (TiO_2_) | (Pd/Cu)^1%^TiO_2_ | Yes | Yes | 0.92 | 60 | 6.6 | 2.6 | 1.3 | >4.0 (LOD) |
| Photocatalytic Peroxone (C_3_N_4_) | (Pd/Cu)^1%^g-C_3_N_4_ | Yes | Yes | 0.92 | 60 | 6.8 | 2.8 | 1.3 | >4.0 (LOD) |

**Table S2.** BET specific surface area, total pore volume and average pore diameter of pristine and bimetallic Pd/Cu modified samples.

| **Sample label** | **BET surface area (m^2^/g)** | **Total pore volume (cm^3^/g)** | **Average pore diameter (nm)** |
| --- | --- | --- | --- |
| TiO_2_ | 10.5 | 0.053 | 20.4 |
| (Pd/Cu)^0.1%^TiO_2_ | 7.8 | 0.042 | 21.8 |
| (Pd/Cu)^0.5%^TiO_2_ | 7.1 | 0.050 | 27.8 |
| (Pd/Cu)^1%^TiO_2_ | 5.6 | 0.070 | 49.8 |
| C_3_N_4_ | 2.9 | 0.047 | 65.9 |
| (Pd/Cu)^0.1%^C_3_N_4_ | 4.3 | 0.063 | 57.8 |
| (Pd/Cu)^0.5%^C_3_N_4_ | 2.3 | 0.053 | 91.9 |
| (Pd/Cu)^1%^C_3_N_4_ | 1.7 | 0.036 | 82.9 |

|  |  |
| --- | --- |
| **Figure S1.**  Structural characterization of different supported Pd/Cu catalysts by SEM, a) (Pd/Cu)^1%^TiO_2_ and b) (Pd/Cu)^1%^C_3_N_4_ photocatalysts. | |

|  | A)   |
| --- | --- |
|  | B)   |
|  |  |
|  | A)   |
|  | B)   |
| **Figure S2.**  Chemical composition of single Pd-Cu particles by HAADF-STEM / EDXS. A), B) and C) indicate the spots where EDXS analysis was performed. | |
|  | A)   |
|  | B)   |
|  | C)   |
|  | A)   |
|  | B)   |
|  | C)   |
| **Figure S2 (cont.).**  Chemical composition of single Pd-Cu particles by HAADF-STEM / EDXS. A), B) and C) indicate the spots where EDXS analysis was performed. | |

**Figure S3.** Nitrogen adsorption-desorption isotherms for a) pristine and bimetallic Pd/Cu modified C_3_N_4_ and b) pristine and bimetallic Pd/Cu modified TiO_2_.

**Figure S4.** LC-OCD organic carbon chromatograms during ozone-based treatments.

**Figure S5.** Ozone inlet and outlet profiles during ozone-based treatments on a) (Pd/Cu)^1%^C_3_N_4_ and b) (Pd/Cu)^1%^TiO_2_ catalyst

**Figure S6.** Bacterial regrowth during photocatalytic ozonation after (left) (Pd/Cu)^1%^C_3_N_4_/O_3_, (right) (Pd/Cu)^1%^TiO_2_/O_3_ treatment
